# Supplementary figures and images for: Hexosamine biosynthesis disruption impairs GPI production and arrests Plasmodium falciparum growth at schizont stages
Source: PLoS Pathog. 2025 Jul 3;21(7):e1012832. doi: 10.1371/journal.ppat.1012832 (PMC12251206; doi:10.1371/journal.ppat.1012832)

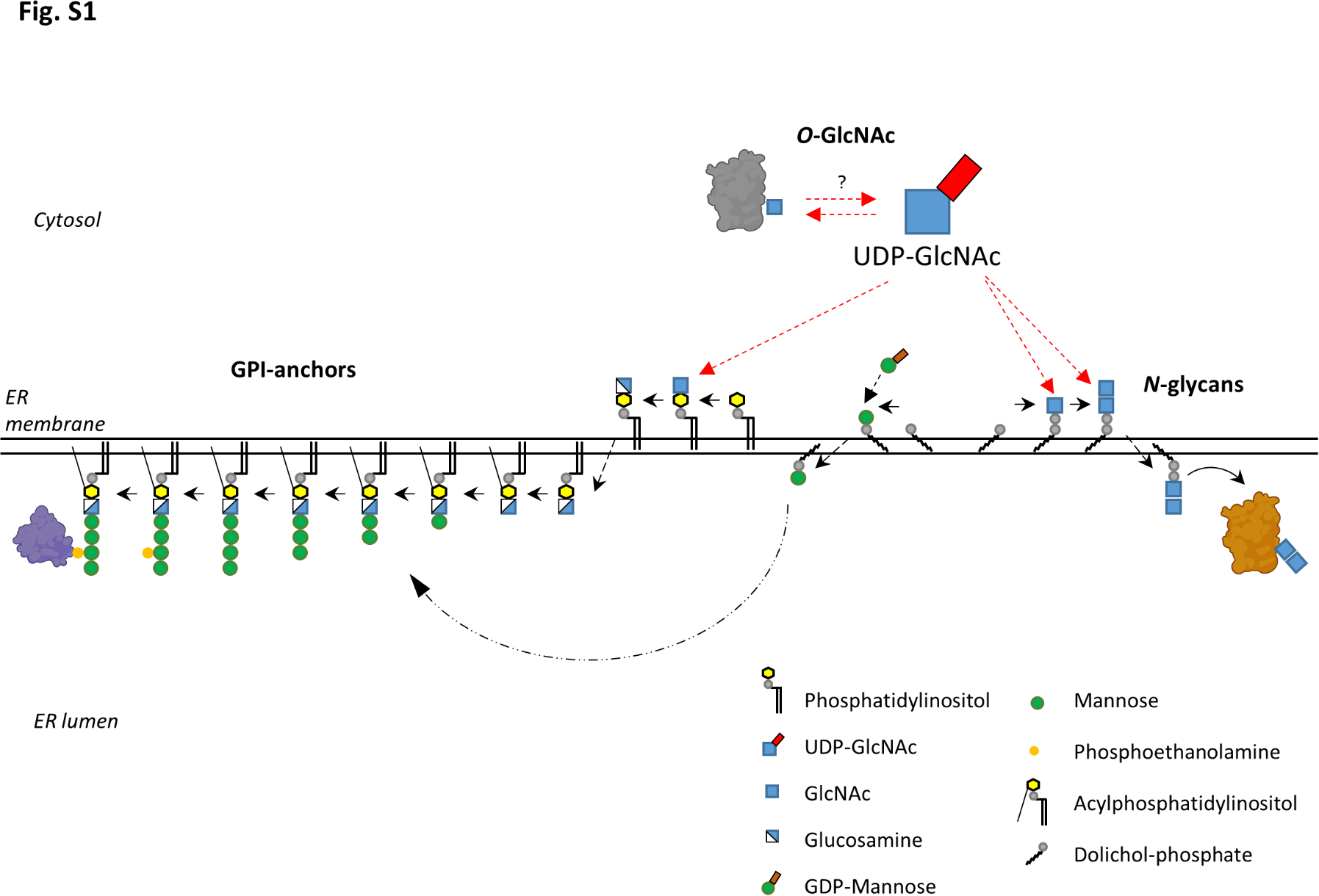

Supplement: S1 Fig — The illustration shows the mechanisms of glycosylphosphatidylinositol (GPI) anchor biosynthesis and N-linked glycosylation in the endoplasmic reticulum. Additionally, it includes the presence of a potential O-GlcNAc cycling, which would require O-GlcNAc transferase (OGT) and O-GlcNAcase (OGA), although these enzymes have not been identified in the P. falciparum genome. Red dashed arrows indicate enzymatic reactions that depend on UDP-GlcNAc, the end product of the Hexosamine Biosynthetic Pathway (HBP). The diagram also includes GDP-mannose, a sugar nucleotide critical for GPI biosynthesis. (TIF) [file ppat.1012832.s001.tif]

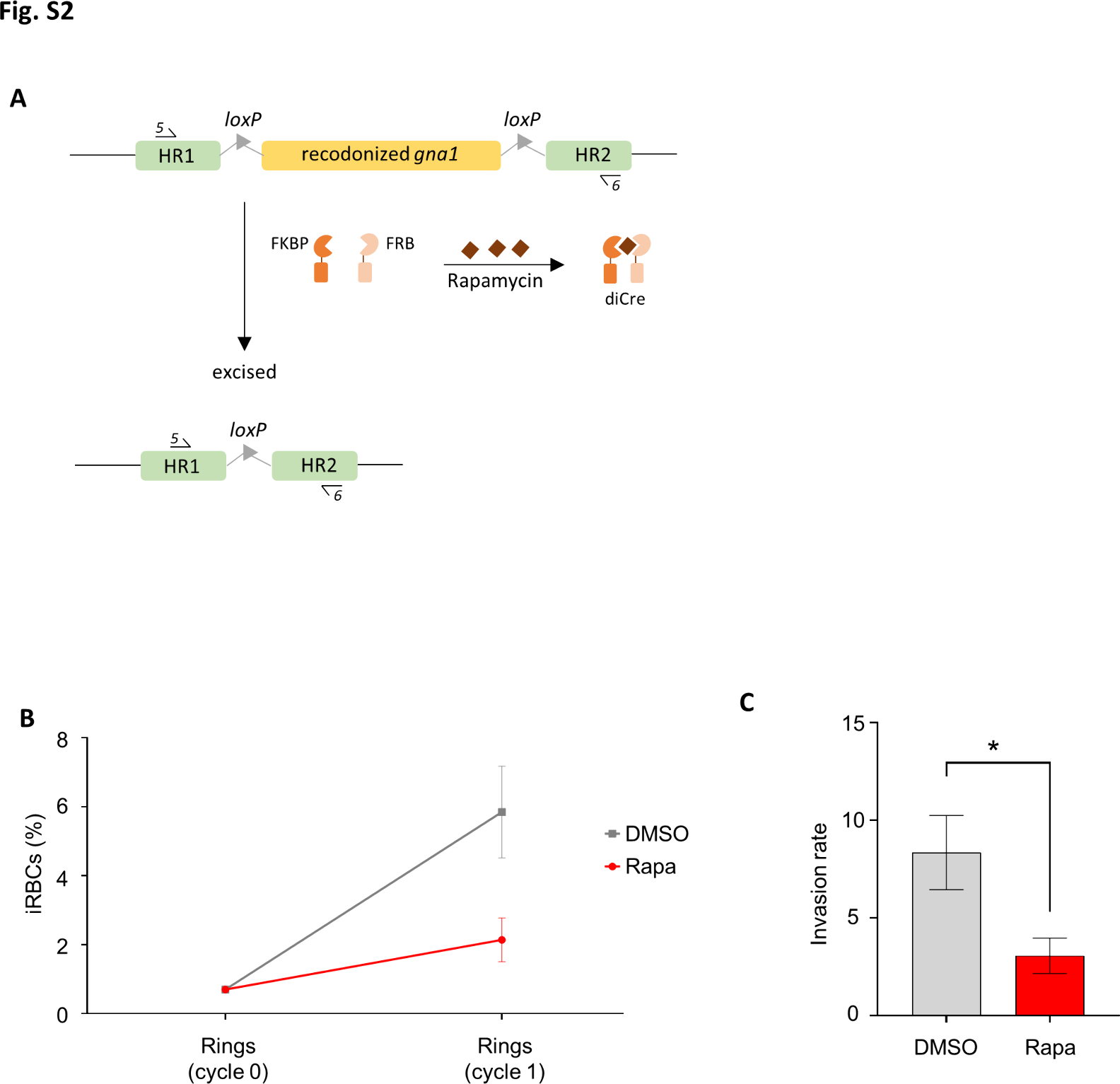

Supplement: S2 Fig — A) Diagram of rapamycin-induced site-specific excision. The transgenic strain II3 gna1-loxP was generated by CRISPR-Cas9 in a DiCre-expressing strain [5]. The native gna1 gene was replaced by a recodonized version gen floxed by two loxP sites. The addition of rapamycin induces Cre recombinase dimerization, which recognizes the loxP sites and removes the sequence between them, inducing gna1 gene excision. Excision reduces the amplicon from 1,546 bp to 738 bp. The homology regions (HR) used for CRISPR-Cas9-based gna1 locus engineering are indicated. The hybridization sites of the primers P5 and P6 used to confirm gna1 excision are also shown. All primers used are described in S2 Table. B) Parasite growth during cycles 0 and 1 following gna1 gene disruption. The II3 gna1-loxP strain was tightly synchronized (5 hours) and treated with rapamycin or DMSO (control) for one hour. Parasitemia was measured immediately (rings, cycle 0) and 60 hours after sorbitol synchronization (rings, cycle 1) by flow cytometry. C) Invasion rates for II3 gna1-loxP parasites treated with either DMSO or rapamycin were calculated for the transition between developmental cycles 0 and 1. In panels B and C the graph shows mean ± SD values of three independent biological replicates. Statistical analyses were performed using unpaired t test. *, P < 0.05; **, P < 0.01; ***, P < 0.001. (TIF) [file ppat.1012832.s002.tif]

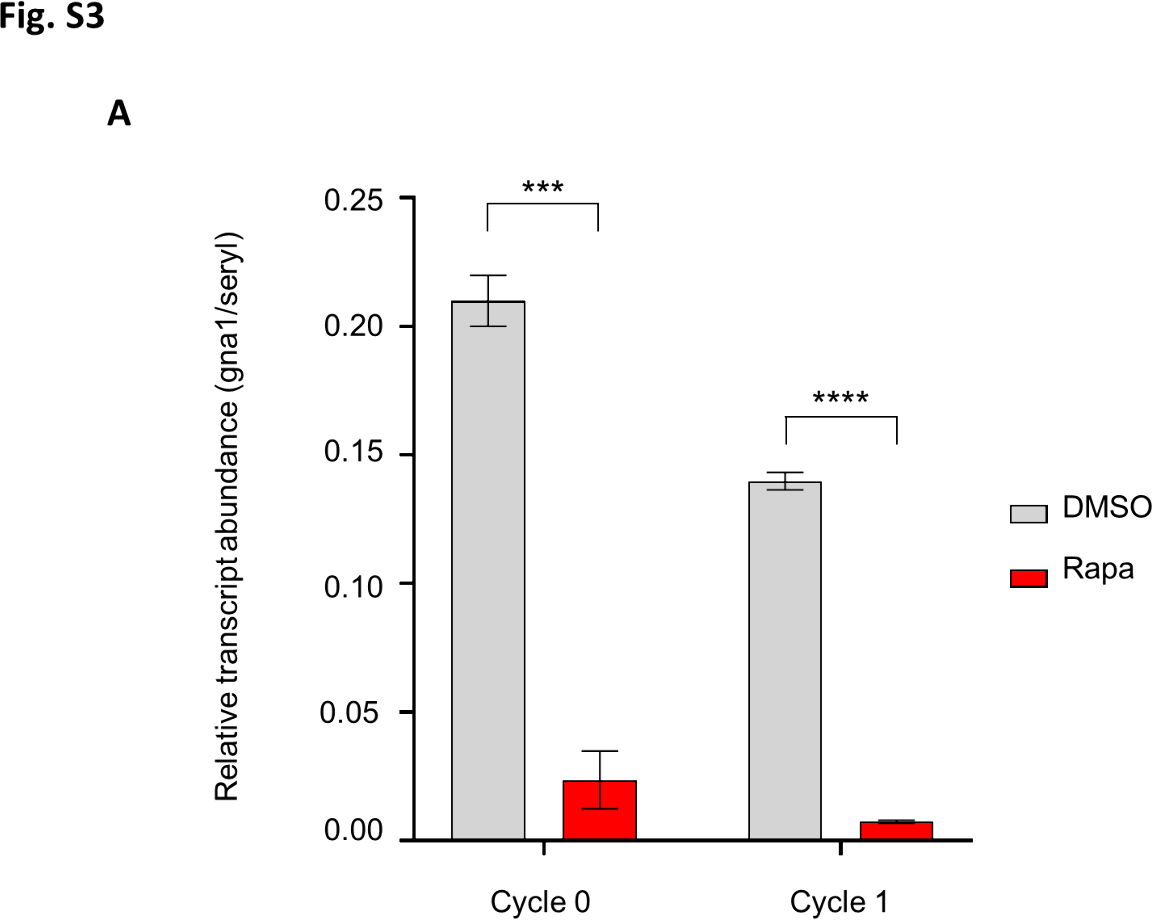

Supplement: S3 Fig — RNAs were extracted from 25-30 hours trophozoites in both, cycle 0 and cycle 1. Transcript abundance was quantified from qPCR data using standard curves and is shown as mean ± standard deviation from three technical replicates. A reduction in transcript abundance was observed in the rapamycin-treated parasites, which is more pronounced in cycle 1 than in cycle 0. Values represent one biological replicate per condition. Statistical analyses were performed using an unpaired t test. ***, P < 0.001; ****, P < 0.0001. (TIF) [file ppat.1012832.s003.tif]

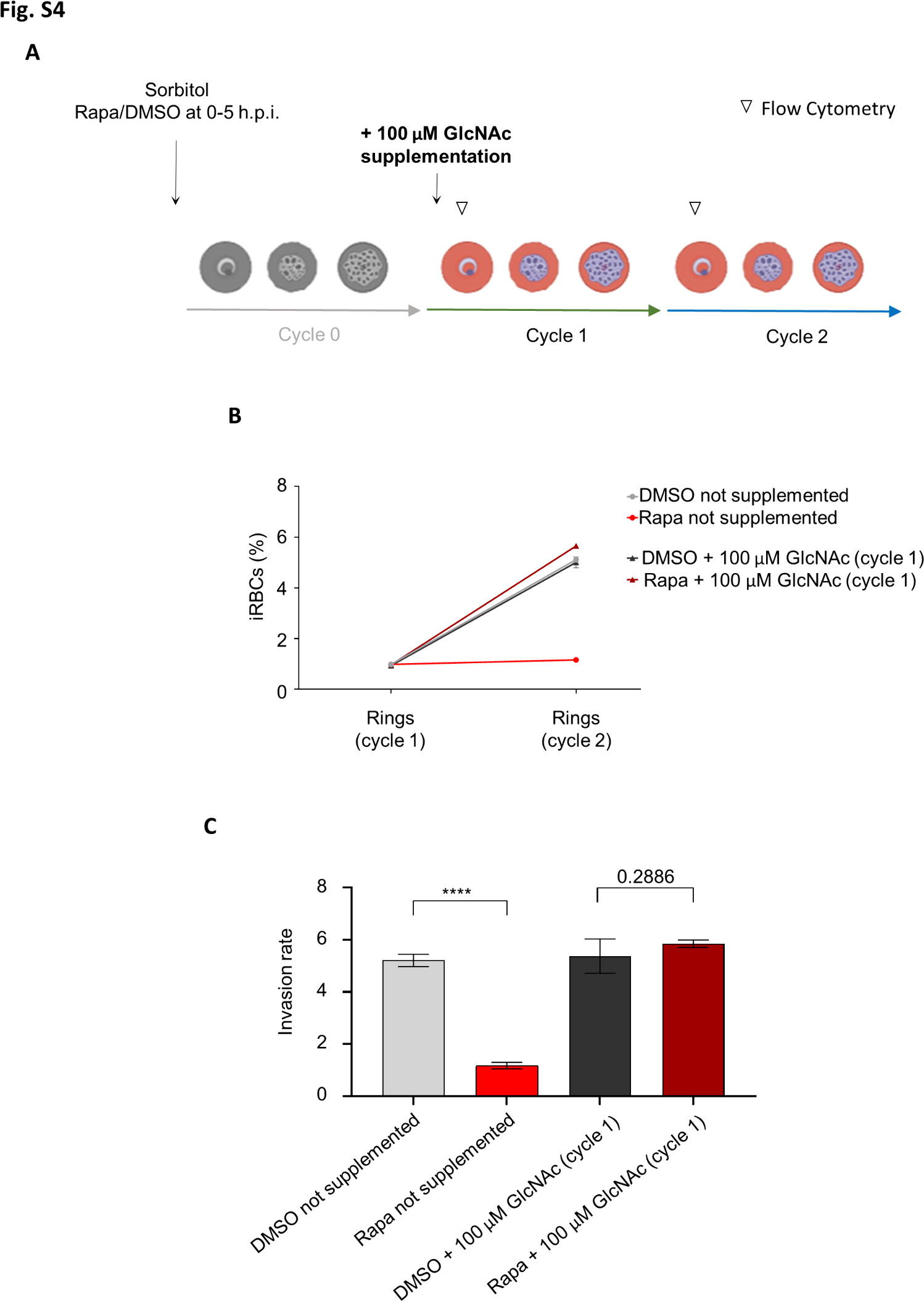

Supplement: S4 Fig — A) Schematic representation of the timing of rapamycin (or mock, DMSO) treatment and GlcNAc supplementation. Rapamycin (or DMSO) were administered for one hour during cycle 0 after tight synchronization of parasites within a 5-hour window. 100 μM GlcNAc was added at the beginning of cycle 1 and maintained until the end of the experiment. The time points at which samples were collected for flow cytometry analysis are also indicated (white arrowheads). B) Parasite growth across cycles 1 and 2 following PfGNA1 disruption, assessed by flow cytometry. C) Invasion rates for parasites treated with either DMSO or rapamycin were measured during the transition from developmental cycle 1–2. In panel C, the graph shows the mean ± SD values of three technical replicates from one representative biological replicate out of three. Statistical analyses were performed using an unpaired t test. ****, P < 0.0001. (TIF) [file ppat.1012832.s004.tif]

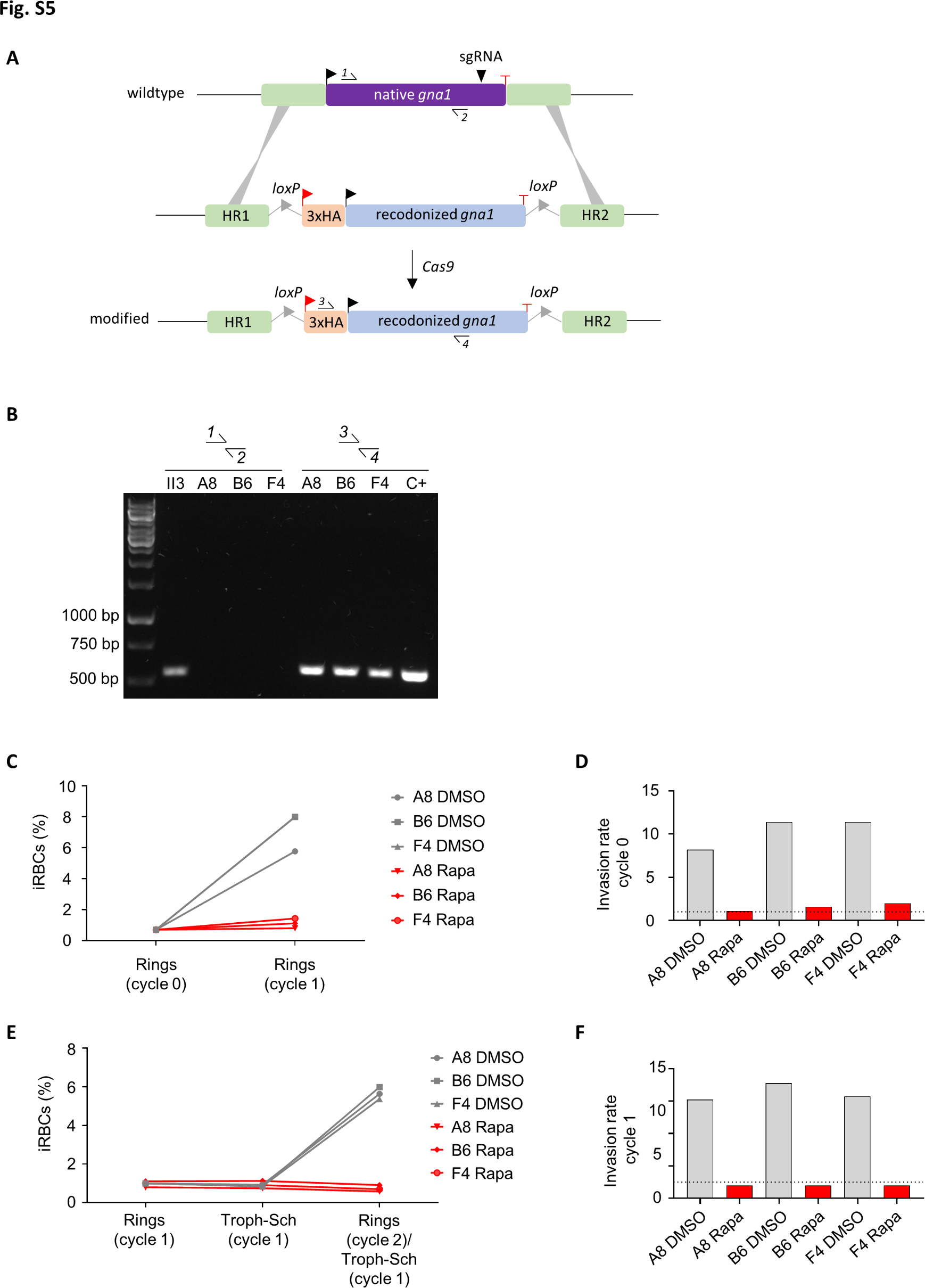

Supplement: S5 Fig — Using Cas9-mediated genome editing, the native gna1 open reading frame was replaced with a recodonized version of the P. falciparum gna1 gene containing three hemagglutinin (HA) epitopes at the N-terminus, with all the insert flanked by two loxP sites. Sequence insertion via double crossover homologous recombination was enabled by Cas9 endonuclease-induced double-strand breaks in the DNA, guided by a single guide RNA (sgRNA) targeting a 20-nucleotide region within gna1. Homology-directed repair was enabled by the addition of homologous sequences flaking the loxP sites. The HA-tag start codon is marked with a red tick mark with an arrowhead. The positions of the start and stop codons for gna1 are indicated by a black tick mark with an arrowhead and a red tick mark with a T-cap, respectively. B) PCR detection of transgenic parasites. PCR-based detection of transgenic parasites was performed using P1 and P2 primers, which amplify a 581 bp of the native gna1. The parental strain (II3) was included as a control. The primers P3 and P4 were used to amplify a 587 bp fragment corresponding to the recodonized version of gna1 in transgenic parasites (Clones A8, B6 and F4). The donor plasmid with the recodonized gna1 sequence, used for the generation of the transgenic lines, was included in the PCR as positive control (C+). All primers used are described in S2 Table. C) Parasite growth during cycles 0 and 1 following gna1 disruption. The II3 gna1-3xHA-loxP strain was tightly synchronized at a 5-hour window and treated with either rapamycin or DMSO (control) for one hour. Parasitemia was assessed immediately (rings, cycle 0) and 55 hours post-sorbitol synchronization (rings, cycle 1) using flow cytometry. D) II3 gna1-3xHA-loxP invasion rates were calculated for the transition between developmental cycle 0 and 1 following treatment with either DMSO or rapamycin. E) Parasite growth during cycle 1 and 2 following gna1 disruption. II3 gna1-3xHA-loxP rings from cycle 1 were adjusted [file ppat.1012832.s005.tif]

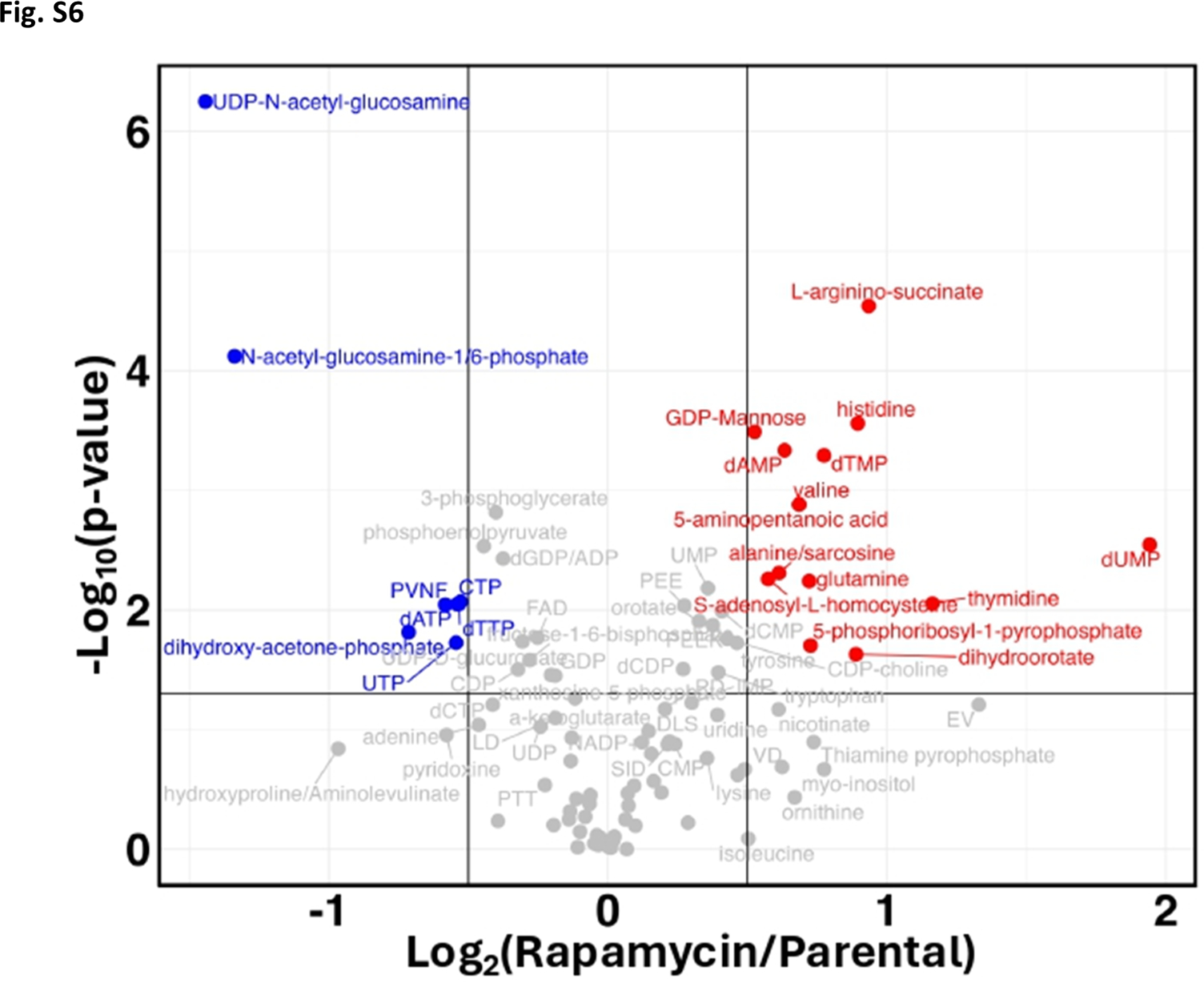

Supplement: S6 Fig — A similar trend is observed when PfGNA1-disrupted parasites are compared with DMSO-treated parasites or parental non-treated parasites. (TIF) [file ppat.1012832.s006.tif]

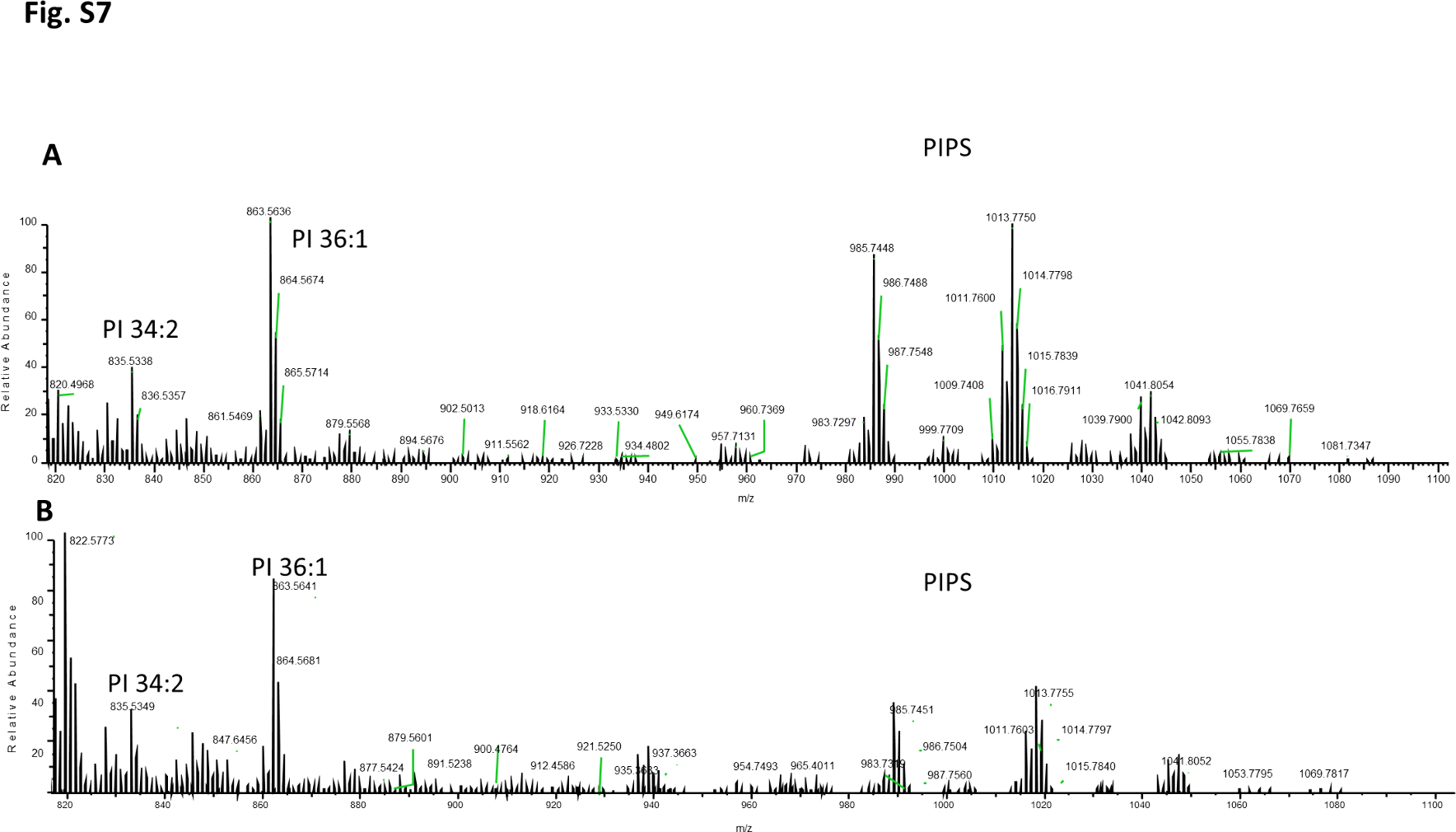

Supplement: S7 Fig — Bligh-Dyer extracted lipids were analysed by ES-MS-MS, negative ion mode 820–1100 m/z, showing primarily the PI and PIP species. A) DMSO and B) Rapamycin PfGNA1-disrupted. Lipid identities confirmed by accurate mass and collision induced fragmentation. (TIF) [file ppat.1012832.s007.tif]

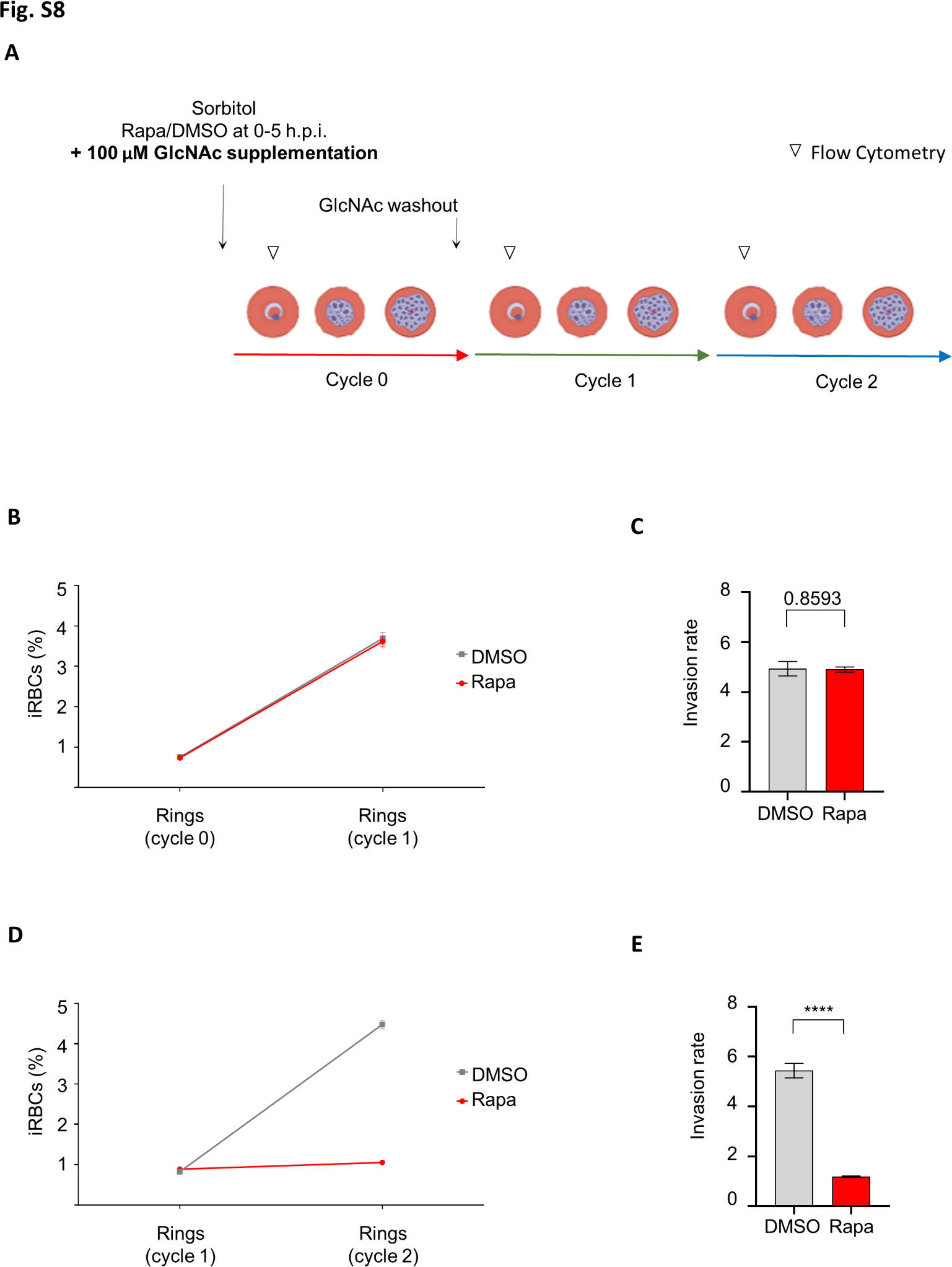

Supplement: S8 Fig — A) Schematic representation of the timing of rapamycin (or mock, DMSO) treatment and GlcNAc supplementation, administered during cycle 0 after tight synchronization of parasites within a 5-hour window. Rapamycin (or DMSO) were added for one hour while 100 μM GlcNAc was added at the beginning of cycle 0 and removed at the end of the same cycle, during the segmented schizont stage. The time points at which samples were collected for flow cytometry analysis are also indicated (white arrowheads). B) Parasite growth across cycles 0 and 1 following PfGNA1 disruption, assessed by flow cytometry. C) Invasion rates for parasites treated with either DMSO or rapamycin were measured during the transition from developmental cycle 0–1. D) Parasite growth across cycles 1 and 2 following PfGNA1 disruption and GlcNAc removal, assessed by flow cytometry. E) Invasion rates for parasites treated with either DMSO or rapamycin were measured during the transition from developmental cycle 1–2. In panels C and E, the graphs show the mean ± SD values of three technical replicates from one representative biological replicate out of three. Statistical analyses were performed using an unpaired t test. ****, P < 0.0001. (TIF) [file ppat.1012832.s008.tif]

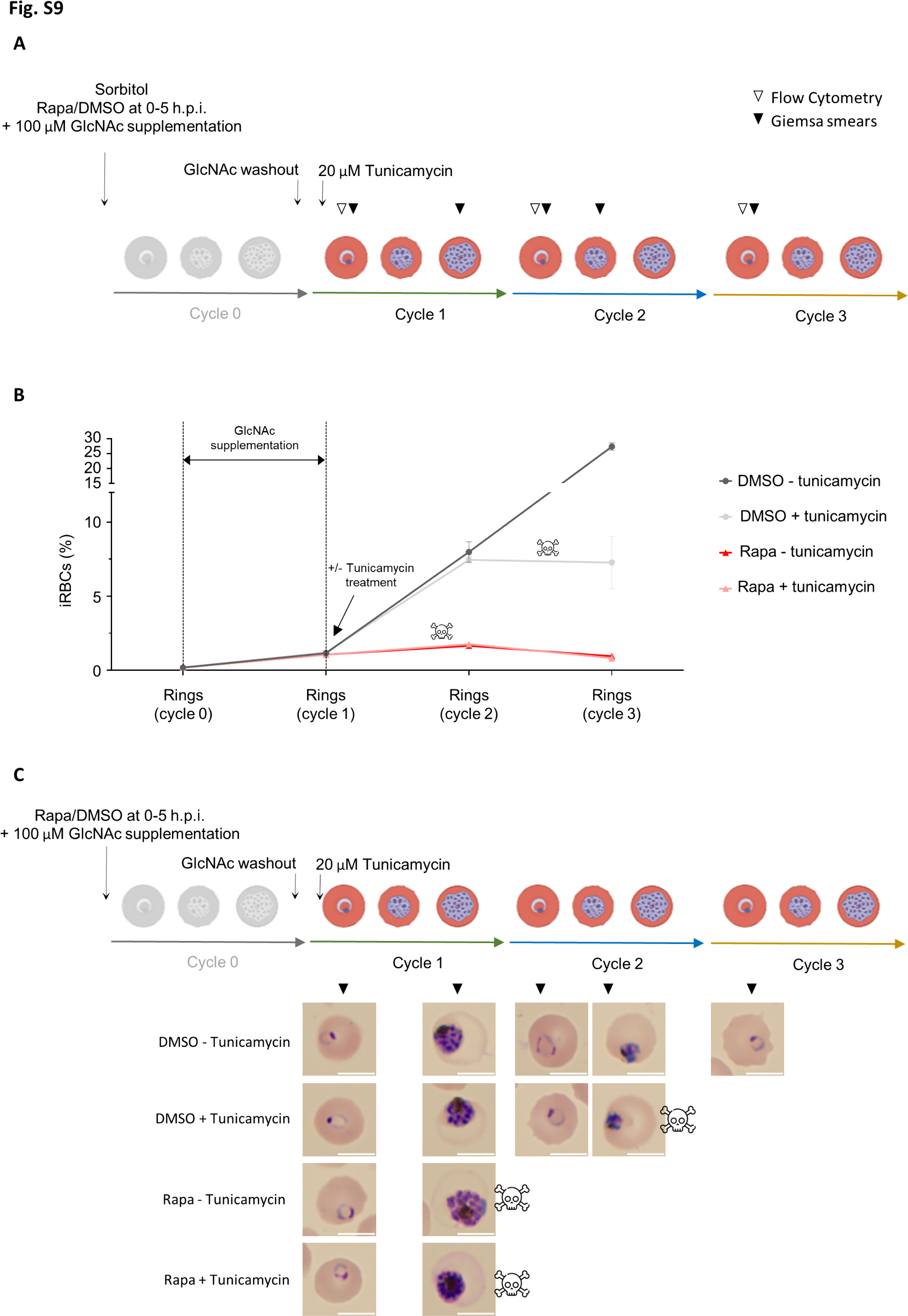

Supplement: S9 Fig — A) Schematic representation of the timing of rapamycin (or mock, DMSO) treatment, GlcNAc supplementation, and tunicamycin treatment. Rapamycin (or DMSO) and GlcNAc were administered during cycle 0 following tight synchronization of parasites within a 5-hour window. Rapamycin and DMSO were added for one hour, while 100 μM GlcNAc was added at the beginning of cycle 0 and removed at the end of the same cycle, during the segmented schizont stage. To dissect the effect of N-glycosylation inhibition on DMSO- (mock) or Rapamycin-treated parasites (PfGNA1- and HBP-disrupted), 20 μM tunicamycin was added at the beginning of cycle 1 and maintained until the end of the experiment. Time points for sample collection by flow cytometry (white arrowheads) and Giemsa staining (black arrowheads) are also indicated. B) Parasite growth from cycle 0 to cycle 3 following PfGNA1 disruption, assessed by flow cytometry. C) Giemsa-stained smear images showing that rapamycin-treated parasites (with or without tunicamycin) failed to progress beyond cycle 1 schizonts, while DMSO-treated parasites exposed to tunicamycin died at the trophozoite stage of cycle 2. Thus, after PfGNA1-disruption parasite death is driven by UDP-GlcNAc depletion which halts GPI biosynthesis, rather than by the effect of tunicamycin. Scale bar represents 5 µm. (TIF) [file ppat.1012832.s009.tif]

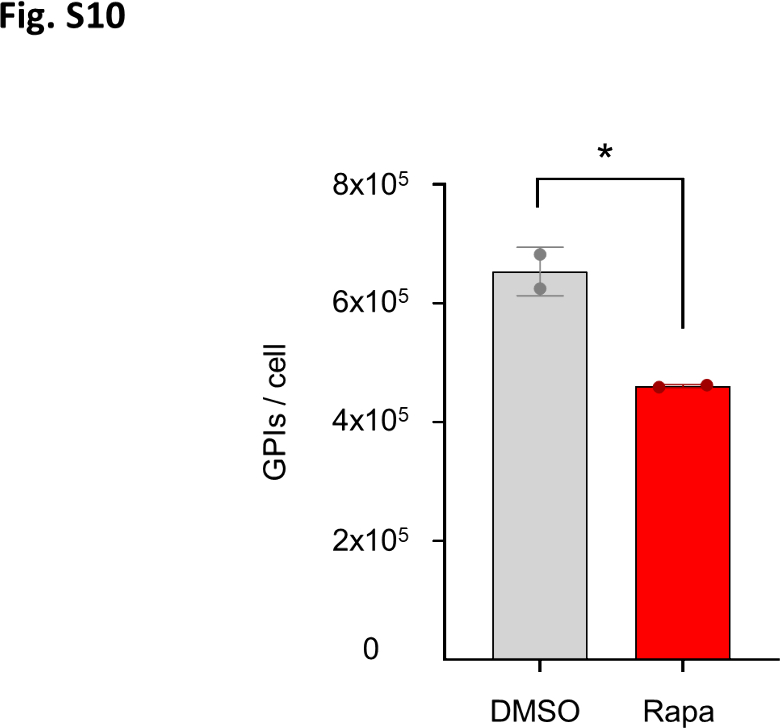

Supplement: S10 Fig — A total of 3.3 × 10⁸ and 2.9 × 10⁸ parasites per replicate were analyzed for the DMSO and Rapamycin conditions, respectively. GPI molecules per cell were also quantified in 3.3 × 10⁸ uninfected RBCs per replicate, and the value obtained was subtracted from the parasite culture values to account for GPI molecules derived from the RBCs. (TIF) [file ppat.1012832.s010.tif]

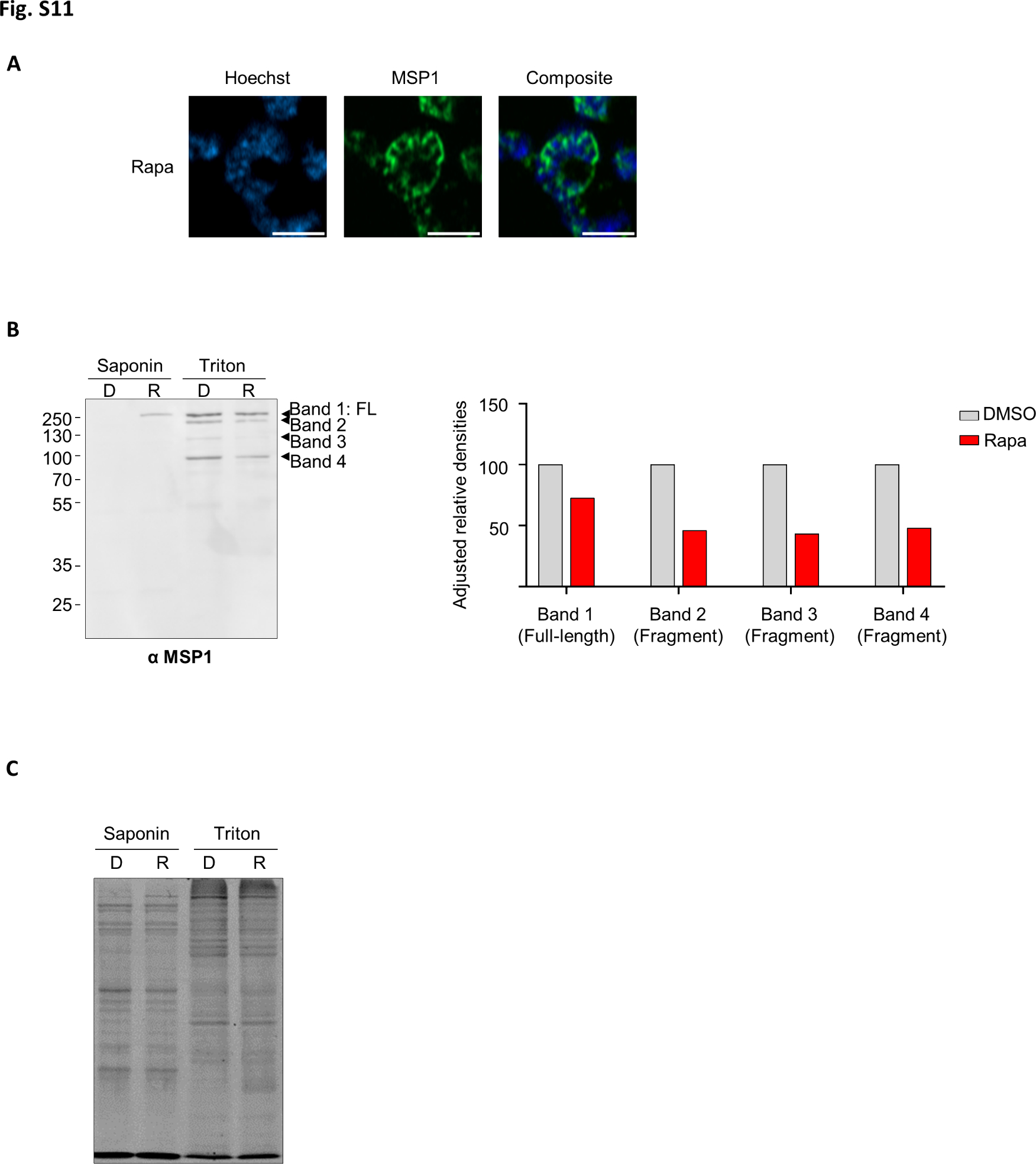

Supplement: S11 Fig — A) Immunofluorescence microscopy showing the typical distribution of MSP1 in less than 6% of PfGNA1-disrupted schizonts. A total of 108 parasites were analyzed. MSP1 was labelled with a mouse anti-MSP1 (green), and nuclei were labelled with Hoechst 33342 (blue). Scale bar represents 5 µm. B) Western blot analysis of MSP1 and quantification of band intensities expressed relative to the control condition, which was set to 100%. Fragments showed a marked reduction in intensity, ranging from approximately 27% to 57% relative to the control. C) Protein bands visualized on the Coomassie-stained gel used as a loading control for the Western blot shown in Fig 3C. A total of 10 μg of protein was loaded for each condition. (TIF) [file ppat.1012832.s011.tif]

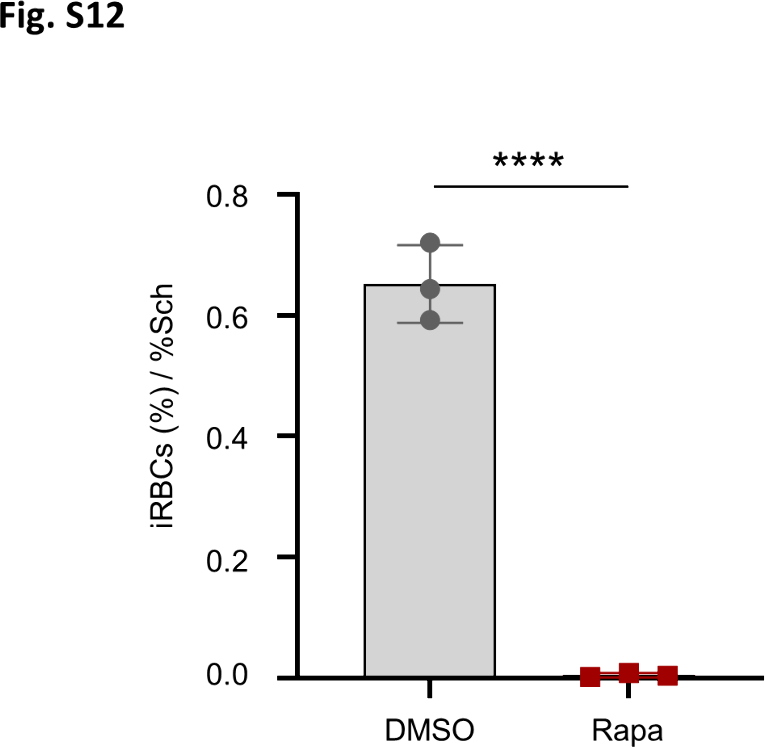

Supplement: S12 Fig — II3 gna1-loxP parasites were treated with rapamycin or DMSO during cycle 0 and incubated until the end of cycle 1. Merozoites were mechanically released from segmented schizonts and subsequently incubated with RBCs to allow invasion. Parasitemia was measured 24 hours later by flow cytometry and reported as the percentage of infected red blood cells relative to the percentage of schizonts before filtration. Statistical analysis was performed using unpaired t test. *, P < 0.05; **, P < 0.01; ***, P < 0.001; ****, P < 0.0001. (TIF) [file ppat.1012832.s012.tif]

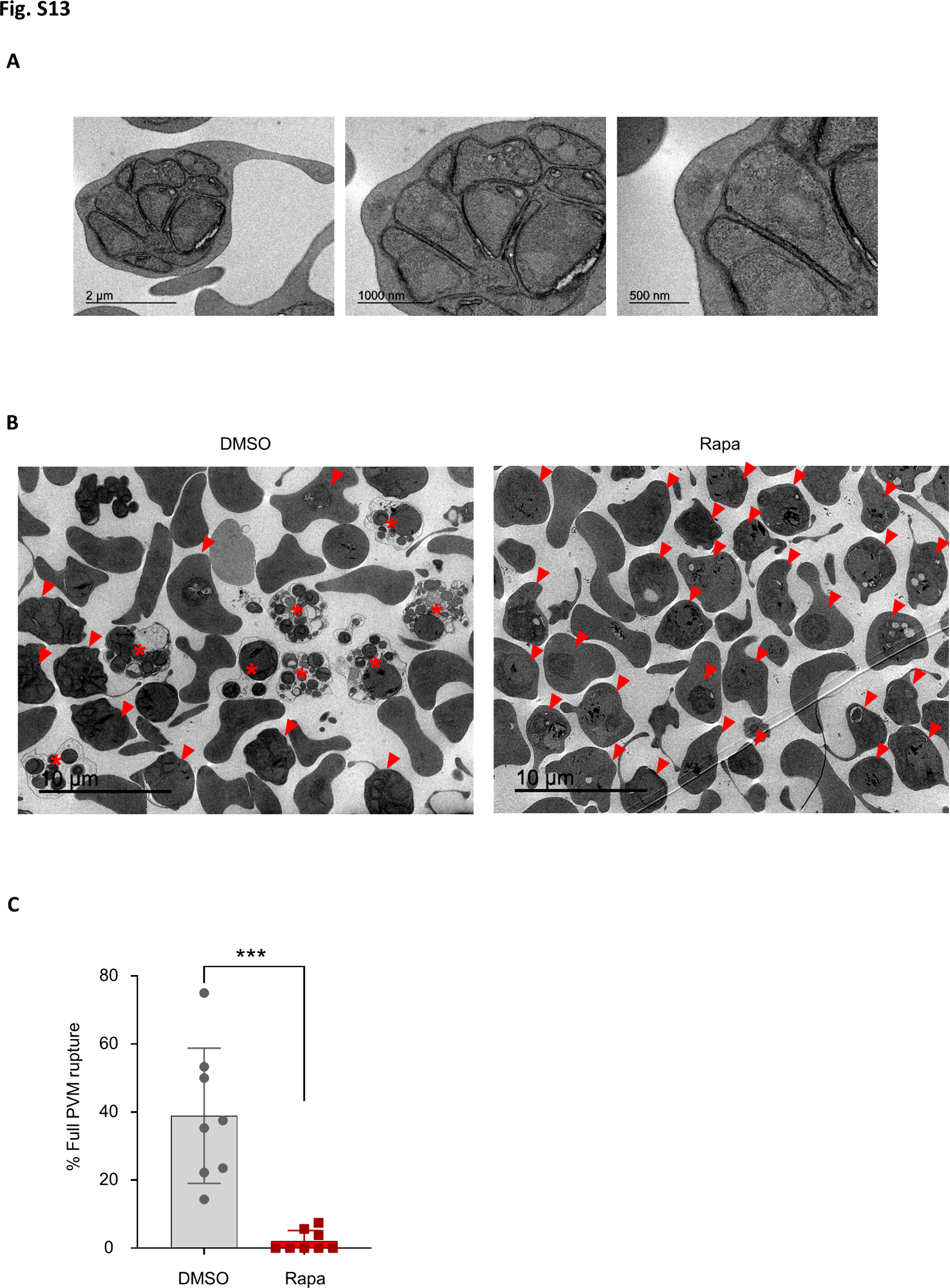

Supplement: S13 Fig — A) Transmission electron microscopy showing normal segmentation in less than 2% of PfGNA1-disrupted schizonts. A total of 90 parasites were analyzed. From left to right, the images were obtained at 30000x, 50000x and 80000x magnification, respectively. B) Transmission electron microscopy images depicting differences in PVM rupture between PfGNA1 conditional knockout schizonts treated with DMSO (control) and rapamycin, following treatment with E64. Arrowheads indicate parasites with an intact PVM, while asterisks mark those with fully disrupted PVM, evidenced by a lighter, less electron-dense appearance of the cytoplasm after PVM breakdown. Magnification: 5000x C) Comparison of the percentage of fully disrupted PVM in PfGNA1 conditional knockout parasites treated with DMSO (control) or rapamycin. Panel B is representative of more than 200 schizonts. For panel C, approximately 15 images containing over 200 schizonts, were analyzed for each condition. Schizonts were categorized based on whether their PVM was intact or disrupted. The statistical analysis of the boxplot in panel C was performed using unpaired t test. ***, P < 0.001. (TIF) [file ppat.1012832.s013.tif]

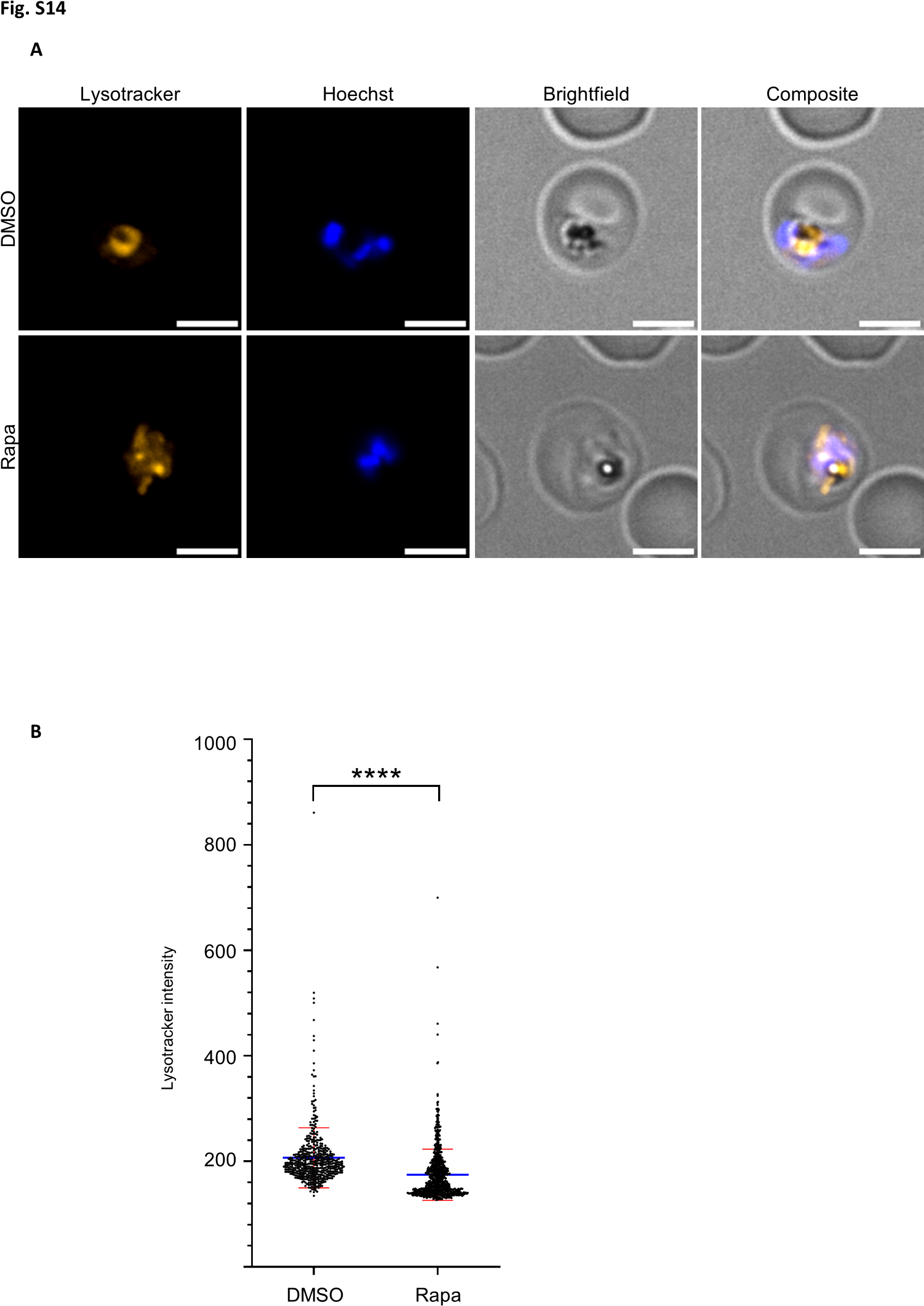

Supplement: S14 Fig — A) Representative immunofluorescence microscopy images showing Lysotracker staining (orange) in segmented schizonts from cycle 1. Nuclei were counterstained with Hoechst 33342 (blue). LysoTracker Red DND-99 is a fluorescent probe that accumulates in acidic compartments. In DMSO-treated parasites, the food vacuole appeared as a distinct, round-shaped organelle with intense fluorescence signal. In contrast, in rapamycin-treated parasites, the fluorescence was dispersed irregularly throughout the cytoplasm. This pattern suggests disruption of the vacuolar membrane and loss of acidic compartmentalization, likely due to the breakdown of the food vacuole structure. Scale bar is 5 µm. B) Quantification of Lysotracker intensity shown as a scatter plot of individual data points. In rapamycin-treated parasites a reduction in LysoTracker signal intensity is observed. The mean is indicated by a blue line, and the standard error of the mean (SEM) by a red line. At least 500 schizonts were analyzed in each condition. Statistical significance was determined using Welch’s t-test. **** P < 0.0001. (TIF) [file ppat.1012832.s014.tif]
