# Supplementary material for: Hexosamine biosynthesis disruption impairs GPI production and arrests Plasmodium falciparum growth at schizont stages
Source: PLoS Pathog. 2025 Jul 3;21(7):e1012832. doi: 10.1371/journal.ppat.1012832 (PMC12251206; doi:10.1371/journal.ppat.1012832)
Supplement: S1 Table — (PDF) [file ppat.1012832.s016.pdf]

Supplementary Table 1. Enzymes involved in the Hexosamine biosynthetic pathway (HBP) in *P. falciparum*

**Enzyme name**	**Enzyme number**	**ID number**[a]	**MFS**[b]
Glucose-6-phosphate isomerase (G6PI)	EC 5.3.1.9	PF3D7_1436000	-3,115
Glucosamine-fructose-6-phosphate aminotransferase (GFPT)	EC 2.6.1.16	PF3D7_1025100	-3,187
Glucosamine-phosphate N-acetyltransferase (GNA1)	EC 2.3.1.4	PF3D7_0629000	-3,365
Phosphoacetylglucosamine mutase (PAGM)	EC 5.4.2.3	PF3D7_1130000	-2,855
UDP-N-acetylglucosamine pyrophosphorylase (UAP)	EC 2.7.7.23	PF3D7_1343600	-3,188

[a] All the gene ID numbers are identified and annotated in the *P. falciparum* genome as putative candidates.

[b] Zhang M, Wang C, Otto TD, Oberstaller J, Liao X, Adapa SR, et al. Uncovering the essential genes of the human malaria parasite Plasmodium falciparum by saturation mutagenesis. Science. 2018 May 4;360(6388):eaap7847
