# Supplementary material for: Hexosamine biosynthesis disruption impairs GPI production and arrests Plasmodium falciparum growth at schizont stages
Source: PLoS Pathog. 2025 Jul 3;21(7):e1012832. doi: 10.1371/journal.ppat.1012832 (PMC12251206; doi:10.1371/journal.ppat.1012832)
Supplement: S2 Table — (PDF) [file ppat.1012832.s017.pdf]

Supplementary Table 2. List of primers used in this study

Name	Sequence
**P1**	AAGAACATTGAACCTATC
**P2**	CTTTATGTATTACAAGTCTTTC
**P3**	CGAAGTTATATGTACCCATACG
**P4**	GATTTGATATCTTCCGCGTT
**P5**	CGCCTTTGTTCATCTCTAAG
**P6**	CGCGAATGGGTTAATCTTTCTACG
**P7**	AAGTATATAATATTGATGTACCTACAAATGTCTATGTTTTAGAGCTAGAA
**P8**	TTCTAGCTCTAAAACATAGACATTTGTAGGTACATCAATATTATATACTT
**P9**	CTGCTGTTTCATATGGCCCG
**P10**	GTCAGGTCACAACGGTTGC
**P11**	AAGTAGCAGGTCATCGTGGTT
**P12**	TTCGGCACATTCTTCCATAA
