# Supplementary material for: Hexosamine biosynthesis disruption impairs GPI production and arrests Plasmodium falciparum growth at schizont stages
Source: PLoS Pathog. 2025 Jul 3;21(7):e1012832. doi: 10.1371/journal.ppat.1012832 (PMC12251206; doi:10.1371/journal.ppat.1012832)
Supplement: Attachment — Submitted filename: Response to Reviewers_PPATHOGENS-D-24-02739.docx [file ppat.1012832.s018.docx]

Editorial Decision from 30.01.2025

Dear Dr.  Izquierdo

Thank you for submitting your manuscript to PLOS Pathogens. After careful consideration, we feel that it has merit but does not fully meet PLOS Pathogens's publication criteria as it currently stands. Therefore, we invite you to submit a revised version of the manuscript that addresses the points raised during the review process.

Please submit your revised manuscript within 60 days Mar 31 2025 11:59PM. If you will need more time than this to complete your revisions, please reply to this message or contact the journal office at [plospathogens@plos.org](mailto:plospathogens@plos.org). When you're ready to submit your revision, log on to <https://www.editorialmanager.com/ppathogens/> and select the 'Submissions Needing Revision' folder to locate your manuscript file.

Please include the following items when submitting your revised manuscript:

* A rebuttal letter that responds to each point raised by the editor and reviewer(s). You should upload this letter as a separate file labeled 'Response to Reviewers'. This file does not need to include responses to any formatting updates and technical items listed in the 'Journal Requirements' section below.

* A marked-up copy of your manuscript that highlights changes made to the original version. You should upload this as a separate file labeled 'Revised Manuscript with Track Changes'.

* An unmarked version of your revised paper without tracked changes. You should upload this as a separate file labeled 'Manuscript'.

If you would like to make changes to your financial disclosure, competing interests statement, or data availability statement, please make these updates within the submission form at the time of resubmission. Guidelines for resubmitting your figure files are available below the reviewer comments at the end of this letter.

We look forward to receiving your revised manuscript.

Kind regards,

Laura Kirkman, MD
Academic Editor
PLOS Pathogens

Tracey Lamb
Section Editor
PLOS Pathogens

Sumita Bhaduri-McIntosh
Editor-in-Chief
PLOS Pathogens
[orcid.org/0000-0003-2946-9497](http://orcid.org/0000-0003-2946-9497)

Michael Malim
Editor-in-Chief
PLOS Pathogens
[orcid.org/0000-0002-7699-2064](http://orcid.org/0000-0002-7699-2064)

**Additional Editor Comments:**

We had the optimal circumstance of multiple reviewers being interested in your paper and as a result were able to obtain five reviews, with differing opinions on what to do moving forward, but also reflecting the general interest in your work. All commented on the quality of the work but several noted that there was concern for the novelty of work given this knockdown line was previously published. Reviewer 5 has suggested some experimental work that would better support your conclusion in this paper that loss of GPI biosynthesis is responsible for the observed phenotype and provide more mechanistic insight.

Thank you for the feedback and the reviewers' insightful comments. We have carefully addressed all concerns, including those regarding novelty, and present new data in the revised version of the manuscript based on the suggested experimental work.

Our response to the editors and reviewers

Dear Laura Kirkman, Tracey Lamb, Sumita Bhaduri-McIntosh and Michael Malim,

We sincerely appreciate the editors and reviewers for their valuable time and effort in conducting a thorough evaluation of our manuscript and for their insightful, constructive feedback. We have carefully addressed each point raised, making revisions to improve clarity, correct minor errors, and refine the presented data. In addition to the specific responses, we have corrected some typographical and minor formatting issues throughout the manuscript; these can be followed in the marked-up copy highlighting all modifications made to the original version. Additionally, we have made several modifications and additions to the supplementary data to further validate and support our findings. Moreover, we present new data that strengthens and significantly expands upon the findings reported in our original submission, providing deeper insight into the underlying mechanisms.

Below, we summarize the major changes made to the presented data:

- We have added new Supplementary Figure S14 using an acidic organelle marker to assess and quantify the integrity of the food vacuole, as suggested by one of the reviewers

- We included new live-cell imaging data further confirming in videos the egress defect in *Pf*GNA1-disrupted schizonts (Supplementary Movie S1). By live cell imaging it was observed that parasites treated with DMSO begin to egress around 14 min after the withdrawal of the egression inhibitor while those treated with rapamycin do not egress, not even after 30 min.

- We used RT-qPCR analysis to further track the dynamics of *gna1* transcript loss following rapamycin treatment, providing a temporal view of gene disruption across cycles (Supplementary Fig. S3).

- We have quantified the total GPI levels in schizonts at the end of cycle 0, demonstrating an early reduction in GPI biosynthesis prior to the more pronounced depletion observed in cycle 1 (Supplementary Fig. S10). This early decrease suggests that GPI biosynthesis is already impacted in cycle 0, supporting a progressive depletion that culminates in the more severe GPI deficiency observed in cycle 1.

- To distinguish the effects of *N*-glycosylation inhibition from those of GPI-anchor biosynthesis disruption, we conducted a series of tunicamycin treatment and GlcNAc supplementation experiments (Supplementary Fig. S8 & S9), supporting the notion that parasite death in cycle 1 is driven by GPI-anchor loss rather than *N*-glycosylation defects.

- We have added rescue assays showing that GlcNAc supplementation in *Pf*GNA1-disrupted parasites rescues the growth phenotype, which reinforces the enzymatic role of PfGNA1 (Supplementary Fig. S4).

- A co-author has been added to the author list: Jonathan Ortega-Barrionuevo. He was critically involved in generating some of the data presented in this revision.

We believe that these significant changes markedly improve our revised manuscript to better align with the publication metrics of *PLoS Pathogens*.

Please find below (in blue) our point-by-point response to the reviewers. Please note that provided pages and lines DO NOT correspond to the clean manuscript version, but to the marked-up copy of the manuscript highlighting the modifications made to the original version (as requested). For clarity, all references cited in this rebuttal are listed at the end of the document following the *PLoS Pathogens* citation style.

**Reviewer #1 - Summary**

The main finding “the disruption of PfGNA1 led to the inability of mature parasites to egress and reinvade new host red blood cells presumably through disruption of the HBP and GPI biosynthesis pathways, thereby halting parasite development.” is novel and relevant.

**Major Issues: Key Experiments Required for Acceptance**

None –

**Minor Issues: Editorial and Data Presentation Modifications**

I suggest adding that disrupted parasite are not able to reinvade new RBC to abstract.

We sincerely thank the reviewer for this suggestion. We had indeed contemplated including this information in the abstract during the initial drafting phase. However, after careful consideration, we decided to focus on the most salient findings of our study. Specifically, the primary defect observed in *Pf*GNA1-disrupted parasites is the developmental arrest at the schizont stage, driven by segmentation abnormalities and lack of parasitophorous vacuole membrane rupture. These defects prevent merozoite release under normal conditions, with only a small number retrieved upon mechanical disruption. While impaired invasion occurs as a secondary consequence, the phenotypic outcome is primarily driven by preceding disruptions in schizogony. To prioritize the central mechanistic findings and maintain clarity, we have chosen to emphasize the upstream defects in the abstract. However, we appreciate the reviewer’s attention to this detail and have now briefly included it in the discussion (page 24, line 521).

Quantification of disrupted or damaged food vacuole seen in 5H would be another killing mechanism important to quantify. You might be able to score existing EM images for rough estimate of disrupted FV like 1% or 10% or more. EM only looks at a single plane and will underestimate. Might a DV membrane protein marker like PfCRT or mdr1 be used to look at the ration of intact DVs. Poosibly a acridine orange stain or another pH marker could quantify percent of ruptured DV with disruption of this pathway.

We appreciate the reviewer’s suggestion. As recommended, we performed staining of cycle 1 schizonts using the acidic organelle marker LysoTracker™ Red DND-99 (Thermo Fisher) to further assess and quantify the integrity of the food vacuole. The first observation from these experiments was a marked change in Lysotracker distribution. In Rapamycin-treated parasites, disruption of the food vacuole likely resulted in altered pH and a diffuse, irregular distribution of the dye throughout the parasite, as observed in immunofluorescence images (see Supplementary Fig. S14A). In contrast, DMSO-treated control parasites retained a well-defined, brightly fluorescent, round-shaped vacuole, consistent with an intact food vacuole. Moreover, Rapamycin-treated parasites displayed a significantly reduced fluorescence intensity compared the control, further indicating disruption of the food vacuole and diffusion of its contents into the cytoplasm (Supplementary Fig. S14B). These new experiments have been included in the revised manuscript (page 17, lines 367-371) and Supplementary Fig. S14.

Line 379 I suggest deleting leading to apoptosis as you have not shown this mechanism. You have other mechanisms of killing. This unsubstantiated mechanism distracts from an substantial body of work.

We have made the suggested changes (page 17, lines 372-373 and page 20, lines 405-406).

Minor

Line 84 suggest “some previous work reports”

We have made the suggested modification (page 3, lines 84-86).

Line 110-please report “may” contribute. The data is weak compared to other pathogen GPI on an inflammatory response. Malaria parasite number 40 million per mL for a one percent parasitemia and the Plasmodium GPI moiety have some but minor contribution to inflammation.

We concur with the reviewer and have made the suggested change (page 4, lines 108-110).

**Reviewer #2 - Summary**

The present study is concerned with the inhibition of the biosynthesis of glycosylinositol, a glycolipid that occurs in free and protein-bound forms in the malaria parasite. The protein-bound form serves as an anchor for proteins in the outer leaflet of a membrane. The free forms play a role in the pathogenicity of malaria. In this respect, the present study could contribute to the development of chemotherapy beyond the mere scientific information.

UDP-GlNAc is required as a component of GPIs for biosynthesis. GlcNac is provided via the hexosamine biosynthesis pathway, which starts from D-glucose. By switching off the enzyme glucosamine-6-phosphate N-acetyltransferase which acetylates GlcN-6-P to GlcNAc-6-P, the biosynthesis of UDP-GlcNac is inhibited and, as a consequence, the synthesis of GPIs.

The investigations are carried out using state-of-the-art methods; this concerns the provision of the mutant, the mass spectroscopic investigations of the lipids and the imaging techniques. The new results are the outcome of collaboration between several highly specialized working groups.

The results merit publication.

**Major Issues: Key Experiments Required for Acceptance**

No absolutely necessary additional experiments.

**Minor Issues: Editorial and Data Presentation Modifications**

This reviewer suggests that the GPI biosynthetic pathway be presented in a similarly compact fashion to that used for the UDP-GlNAc biosynthetic pathway in Fig. 1A.

This would be helpful for readers outside the GPI domain.

We acknowledge the reviewer’s suggestion and recognize the value of presenting the GPI biosynthetic pathway in a more concise manner. This was carefully considered during manuscript preparation. However, given GNA1’s central role in the Hexosamine Biosynthetic Pathway and the potential diversion of UDP-GlcNAc into alternative pathways, we aimed to keep Fig. 1A as clear as possible. To ensure that the figure panel remains clear and focused, we elected to present the GPI biosynthesis, *N*-glycosylation, and *O*-GlcNAcylation pathways in Supplementary Fig. S1. In addition, we have now highlighted the ER membrane in Supplementary Fig. S1 to clarify the cellular compartments involved.

The authors show by imaging techniques that the distribution of GPI-anchored proteins is abnormal compared to normal conditions. In my opinion, further clarification is needed. Is it caused by structural deviations of the GPI parts of the proteins? Are they proteins before transmission of the anchor that are anchored via their non cleaved hydrophobic end in the membrane? This could be clarified in part with the help of phospholipases or glycosidases.

In any case, the authors should at least include these aspects in their discussion.

We sincerely appreciate the reviewer’s insightful comments. MSP1, the most abundant GPI-anchored protein on the merozoite surface, serves as a reporter in our study. Our data indicate that in *Pf*GNA1-disrupted parasites, MSP1 localization is disrupted, which coincides with a major decrease in GPI moieties. However, as noted in the main text (page 10, lines 247–248) and Supplementary Fig. S11, a subset of parasites still retains a typical MSP1 distribution. This suggests that at least some of the protein detected in the Triton X-100 fraction of rapamycin-treated parasites (Fig. 3C) remains membrane-bound via GPI anchors. As noted by the reviewer, this fraction may also contain proteins associated with the ER membrane, due to strong interactions with membrane-bound proteins or lack of processing by the GPI-transamidase complex, likely resulting from the depletion of GPI molecules as anchor acceptors.

To further explore this, we performed a digestion using GPI-specific phospholipase D (PI-PLD) on saponin-treated parasite pellets, followed by Western blot analysis, using anti-MSP1. In DMSO-treated control parasites, PI-PLD efficiently released MSP1—mostly in its processed form (lower molecular weight)—into the supernatant. In rapamycin-treated parasites, MSP1 was also released, both in its processed and unprocessed (higher molecular weight, precursor) form. Notably, MSP1 remained detectable, both processed and unprocessed, in the Triton X-100 fraction under both conditions. This suggests incomplete PI-PLD cleavage, residual membrane association and/or retention within intracellular secretory compartments, potentially via non-GPI mechanisms such as insertion through uncleaved hydrophobic domains. These findings are consistent with the possibility that both GPI-anchored and non-GPI-associated forms of MSP1 may coexist under conditions of impaired GPI biosynthesis. Given that the outcome of this experiment is not entirely conclusive, we chose to include the figure in our response solely to demonstrate the experimental effort to the reviewer, but opted not to incorporate it into the manuscript, either as a main or supplementary figure.

Parasites were treated with DMSO or rapamycin, and at the schizont stage, parasitophorous vacuole contents were released using saponin (left). Parasite pellets were then digested with 2.5% fetal bovine serum containing phospholipase D (PLD, center). Finally, samples were extracted with Triton X-100 (right), and both supernatant and pellet fractions were analyzed by SDS-PAGE and Western blotting using anti-MSP1.

Interestingly, a recent study by Bulloch et al. (2024, *PLoS Pathogens*) reported a similar phenotype in parasites with impaired GPI biosynthesis due to Dol-P-Mannose depletion [1]. They demonstrated that a portion of the MSP1 proteins retained in the detergent fraction were indeed GPI-anchored, aligning with our observation that some parasites maintain MSP1 localization. However, they also found that some of these proteins were resistant to GPI-anchor cleavage, which could support the reviewer’s hypothesis.

In light of this, we have incorporated these findings into the discussion section, as suggested (page 22, lines 469-473).

**Reviewer #3 - Summary**

In this manuscript, Alberione et al. have characterized the hexosamine biosynthesis pathway and discovered that PfGNA1 is essential for GPI production, parasite egress and viability. Overall, the study was well designed and supported by good data. There are several issues that should be addressed.

**Major Issues: Key Experiments Required for Acceptance**

1, Throughout the paper, the authors refer to the first experimental cycle as cycle 0, which is somewhat misleading. It should be cycle 1 as it indicates the first cycle. The authors need to adjust all the cycle numbers accordingly to avoid any confusion (although the authors’ prior publications used cycle 0 to indicate cycle 1).

We appreciate the reviewer’s concern regarding the cycle numbering and acknowledge the potential for confusion. However, we have adopted the 'cycle 0' and 'cycle 1' nomenclature to distinguish between the cycle in which rapamycin induction and gene ablation occur (cycle 0) versus the subsequent cycle where the full phenotypic consequences of gene disruption manifest (cycle 1). This convention has been used previously in several studies [2–9] to account for the dynamics of gene ablation, including the persistence of residual protein in cycle 0, which can temporarily sustain parasite development. In the case of *Pf*GNA1, because a dynamic pool of UDP-GlcNAc is already present, this effect is even more pronounced, resulting in only a marginal impact on parasite growth during cycle 0 (see Supplementary Fig. S2). The most pronounced defects emerge in cycle 1, and include developmental arrest and loss of viability. In our previous work [9], we focused primarily on determining whether the enzyme is essential for parasite survival and characterizing its enzymatic function, rather than analyzing the phenotypic consequences of *Pf*GNA1 ablation in detail. Given the in-depth phenotypic analysis conducted in the current study, we believe this nomenclature provides greater clarity. To prevent confusion, we have also explicitly clarified this distinction in the results section (page 5, lines 138-146).

2, In addition to TEM, the egress defects should be better illustrated using live cell imaging. If this technique is not immediately available, the authors need to discuss this and indicate the limitations of TEM such as fixation artifacts and etc.

We agree with the reviewer’s suggestion regarding the use of live cell imaging to further illustrate the egress defects in *Pf*GNA1-disrupted parasites. To address this, we have now performed live cell imaging analysis and included representative videos as supplementary information (Supplementary Movie S1) to further support our findings. We have also revised the manuscript text (page 13, lines 293-295). In Fig. 4B, Giemsa-stained smears reveal the persistence of schizonts more than 15 hours after egress has occurred in DMSO-treated parasites. Similarly, in Fig. 4C, over 80% of schizonts remain arrested for at least 6 hours, whereas in DMSO-treated parasites, fewer than 20% persist. Finally, by live cell imaging we observed that parasites treated with DMSO begin to egress around 14 min after the withdrawal of the egression inhibitor while those treated with rapamycin do not egress, not even after 30 min. Combined, these new imaging approaches corroborate the egress inhibition seen in the rapamycin-treated parasites.

We agree with the reviewer that while TEM provides valuable ultrastructural insights, potential fixation artifacts must be considered when interpreting these findings. However, the absence of aberrant structures in DMSO-treated parasites, along with the persistent schizont arrest observed in Fig. 4B and 4C and by live-cell imaging supports the idea that these defects are biologically relevant rather than an artifact of sample preparation.

3, The idea that the inducible knockout (iKO) parasite was undergoing apoptosis has not been fully supported by the data. Nuclear condensation was not evident in Fig 5G/J. Other features of ‘apoptosis’ were not investigated.

We appreciate the reviewer’s comment and agree that the evidence for programmed cell death in *Pf*GNA1-disrupted parasites is not fully conclusive. Although chromatin condensation is often associated with apoptosis, it can also occur in other types of cell death. To address this, we have revised the text to clarify that while some observed features hint at cell death-like traits, further investigation is needed to dissect the specific type of cell death that is occurring (page 17, lines 371-381 & page 20, lines 405-406).

4, One of the most striking features of the iKO was shown in Fig5, but it was not fully discussed by the authors. iKO seems to have more ribosomes, a phenomenon not just derived from irregular distribution. The authors focused on GPI and GPI-anchored proteins (MSP1) in this study. They should also check the literature to see whether anything is known about hexosamine biosynthesis and ribosome biogenesis.

We appreciate the reviewer’s insightful comment regarding ribosome abundance in *Pf*GNA1-disrupted parasites and the suggestion to explore a potential link to hexosamine biosynthesis. While our study primarily focused on UDP-GlcNAc production and its downstream effects on glycoconjugate synthesis, we acknowledge that broader metabolic changes caused by HBP perturbation could influence ribosome biogenesis. Although there are some indirect links between the HBP, nutrient sensing, glutamine metabolism, and cell growth regulation, to our knowledge current literature does not establish a direct connection between the HBP and ribosome biogenesis [10,11]. Given this, we have chosen to avoid overinterpretation and therefore have not expanded on this aspect in the current manuscript.

The citation #40 was not properly used here as the study showed ribosome reduction after drug treatment, which is a totally different phenomenon than ribosome increasement upon knockout of GNA1.

We acknowledge this comment and have modified the text accordingly (page 17, lines 375-381).

**Minor Issues: Editorial and Data Presentation Modifications**

1, Fig 1A. Please illustrate the structures of the molecules in the HBP pathway.

We appreciate the reviewer’s suggestion. The structures of the molecules in the HBP pathway are now included in Fig. 1A.

2, Fig 1E. Please clearly describe how invasion rate was calculated. Was it the ratio of ring% compared to schizonts% of the prior cycle?

The invasion rate was calculated as the ratio of newly infected RBCs (iRBCs) to the iRBCs from the previous cycle. While this largely corresponds to the ratio of rings to schizonts, it more generally accounts for iRBCs at different stages depending on the sampling point. We have now clearly included this information in the Materials and Methods section, under ‘*P. falciparum* growth analysis’ (page 26, lines 564-565).

3, Fig 1F. Please comment on the protein expression level of GNA1. How much protein was loaded? The band ~ 30 kDa is barely visible. There is a more abundant band around 10 kDa. Was it a degradation product or a partially cleaved product of GNA1?

We agree with the reviewer that the GNA1 band (~30 kDa) is faint in Figure 1F, despite loading 10–15 µg of total protein per lane. We tried to extensively optimize the experimental conditions, including testing multiple anti-HA antibodies, varying protein extraction buffers, and adjusting transfer/incubation parameters. Unfortunately, these efforts did not improve the signal intensity. The ~10 kDa band was reproducibly observed under identical conditions in replicate experiments, but its identity remains unclear. While it may represent a degradation or cleavage product, we lack definitive evidence to confirm this. Notably, auto-adjusting brightness and contrast in ImageJ makes the reduction of the ~30 kDa band in rapamycin-treated parasites readily apparent. We have now modified panel 1F to show this.

4, Line 126, the correct citation should be #5, not #6.

We thank the reviewer for identifying this oversight. The citation on Line 126 (page 5) has been corrected to #5 in the revised manuscript.

5, Line 233. The authors should indicate how many parasites were investigated and what is the percentage of normal MSP1 distribution in the inducible KO.

Only 6 rapamycin-treated parasites out of 108 investigated (less than 6%) showed a seemingly normal segmentation in 63x immunofluorescence images. We have now included these data in the legend of Supplementary Fig. S11A.

6, Fig 3C. In blots with MSP1 and AMA1, please indicate which band is the full-length protein and what are the bands of cleaved (or processed) products.

We thank the reviewer for this suggestion and have updated Fig. 3C to indicate the unprocessed 196 kDa band and the processed fragments. As shown, the saponin fraction of rapamycin-treated parasites contains the full-length unprocessed form. Notably, the processed fragments in the Triton X-100 fraction appear less intense, suggesting reduced processing in *Pf*GNA1-disrupted parasites, possibly due to the absence of GPI anchoring, which likely affects a major fraction of MSP1 proteins. Fig. 3C legend (page 12, lines 279-281) and the text (page 11, lines 259-263) have been modified accordingly.

7, Line 246. This statement should be supported by quantification of band intensities. Fig 3C showed the bands in the Triton/Rapamycin lane were fainter compared to Triton/DMSO. It remains unclear whether MSP1 processing was affected.

We thank the reviewer for this observation. We have now clarified this point in the text, in line with the modifications made to Fig. 3C, as noted in our previous response (page 11, lines 259-263). We have also calculated the relative intensities of the MSP-1 bands in Triton X-100 lanes to further support our assumption (Supplementary Fig. S11B).

8, Line 280. This citation was not numbered.

Again, we thank the reviewer for spotting this error, and we have included the proper citation in the revised manuscript (page 13, line 299).

9, Line 323. The authors need to indicate how many parasites were investigated and what is the percentage of normal segmented parasites in the inducible KO.

Only 1 rapamycin-treated parasite out of 90 showed seemingly normal segmentation in 5000x TEM images. We have now included these data in the legend of Supplementary Fig. S13A.

10, Line 379. Apoptosis was not fully verified in this study.

We acknowledge the reviewer’s comment. This point has been addressed in our responses to Reviewer #1 and incorporated into the revised text (page 17, lines 371-373 & page 20, lines 405-406).

11, Supplementary Typos, Line 54, y F4

Thank you, we have now corrected this mistake (Supplementary Fig. S5B)

12, Fig S3A, please indicate the positions of the start and stop codons.

We have addressed this suggestion by indicating the positions of the start and stop codons in the *gna*1 gene (Now Supplementary Fig. S5A)

**Reviewer #4 - Summary**

It is well established that Plasmodium has limited carbohydrate modifications to its proteins, and an apparent majority of the UDP-GlcNAc pool is used in the synthesis of GPI, which tethers a number of surface proteins to the parasite plasma membrane. In the present study, the authors phenotypically assess the inducible knockout of PfGNA1, which they have previously shown to be essential for parasite replication, presumably through its role in the hexosamine biosynthesis pathway. While the experiments performed here appear to be of high quality, new mechanistic insight is limited.

The authors have already published that inducible deletion of GNA1 blocks parasite replication through the expected biochemical mechanism. They have nice microscopy that shows mislocalization of one GPI-anchored protein (MSP1, which is known to be essential). They also have nice EM showing parasites are nonviable by the time of egress. But given the long time period in assessing phenotype (a full lytic cycle), and the fact that GPI anchored proteins like MSP1 are known to be essential, there isn’t much mechanism to be had here that wasn’t already known. The authors note that a large portion of the GPI in membranes is not anchored to any protein, and suggest it may be required for egress/motility. But no effort was made in the present study to differentiate whether the phenotype is due to a lack of GPI-anchored protein or just free GPI (probably both; I understand that this would be quite difficult to ascertain).

We thank the reviewer for their thoughtful comments and for acknowledging the quality of our microscopy and EM work. However, we respectfully disagree with the notion that our study lacks new mechanistic insight.

In our previous studies, we uncovered and characterized an apicomplexan-specific GNA1 enzyme, confirming its enzymatic activity across species [12] and highlighting its apicomplexan-specific structure and striking differences from the human ortholog [9]. Given these differences, we demonstrated that GNA1 plays an essential role in parasite proliferation, suggesting its potential as a target for selective inhibition of parasite growth [9]. However, these studies did not investigate the specific downstream effects of *Pf*GNA1 disruption. In contrast, our present manuscript provides key insights into 1) how HBP disruption impacts UDP-GlcNAc levels, 2) how this in turn majorly affects GPI-anchor, and ultimately 3) how this alters and disrupts parasite development throughout the asexual blood stages. Additionally, while the reviewer states that “*the authors have already published that inducible deletion of GNA1 blocks parasite replication through the expected biochemical mechanism*,” the exact mechanism was not previously known. It remained unclear whether the phenotype was driven mainly by GPI depletion or if other pathways were also affected and contributed to parasite growth defects. Our new study directly addresses this knowledge gap. Furthermore, our work suggests that parasite death occurs within one asexual life cycle, despite some delay likely due to the specific dynamics of our inducible knockout system. This finding has important implications for considering the HBP pathway and GPI biosynthesis as potential sources of new therapeutic targets against the asexual blood stages of the malaria parasite.

Although GPI-anchored MSP1 is critical for *P. falciparum* survival, *Pf*GNA1-disrupted parasites exhibit defects not only in invasion (or egress) but also in segmentation and PVM rupture. While GPI anchors were assumed to be essential for parasite growth, their specific role in these processes had not been conclusively shown. Notably, a recent independent study linking apicoplast dysfunction to GPI depletion via dolichol-phosphate deficiency reported a strikingly similar phenotype [1]. However, our findings clarify the mechanism by directly linking these defects to GPI anchor biosynthesis disruption, rather than to broader changes in lipid metabolism, such as alterations in cis-polyisoprenol and dolichol pools. Thus, our results provide a relevant confirmation of the critical role of GPI anchors in parasite survival, refining the mechanistic understanding of parasite death following HBP disruption and UDP-GlcNAc depletion.

As noted by the reviewer, differentiating whether the phenotype arises from the lack of GPI-anchored proteins or free GPI—or both—is difficult and beyond the scope of this study and our capabilities at the moment, although we are designing future studies to ascertain this. Nevertheless, the striking decrease in the level of total GPI molecules in rapamycin-treated cells (assembling only 10% of the GPI molecules in comparison to DMSO-treated parasites) suggests that both GPI-anchored proteins and GPI anchors are severely affected.

We hope that the revisions made in response to all reviewers' comments, along with our detailed responses, will demonstrate the quality and significance of our new studies. We trust that these clarifications will address the reviewer’s concerns and lead to the manuscript's acceptance for publication.

**Major Issues: Key Experiments Required for Acceptance**

None –

**Minor Issues: Editorial and Data Presentation Modifications**

None –

**Reviewer #5 - Summary**

This study investigates the consequences of disrupting hexosamine biosynthesis (specifically GlcNAc6P synthesis) in the malaria parasite, Plasmodium falciparum. Rapamycin inducible knock-down of the gene encoding GlcN6P acetyltransferase (GlcN6P-AT) in asexual blood stages leads to parasite death over 1-2 cycles of infection, and correlates with a reduction in intracellular levels of down-stream intermediates in the hexosamine biosynthesis pathway; GlcNAc1/6P and UDP-GlcNAc. Further, disruption of the pathway leads to significant ultrastructural changes in blood stages, defects in the processing of the major GPI-anchored protein, MSP1, and defects in parasite egress. The authors propose that the observed phenotype primarily reflects the loss of GPI biosynthesis and expression/processing of GPI-anchored proteins, rather than loss of other GlcNAc-dependent pathways such as protein N-glycosylation. There is clear interest in understanding the function of different glycosylation pathways in these parasites and the work described in the manuscript is generally well performed. However, the novelty of this study is unclear. The authors have recently published a paper in which the same enzyme is inducibly knocked-out in the same parasite (Ref 5,6), and several other studies have shown that enzymes/pathways involved in GPI biosynthesis are essential in these parasites. More importantly, while the study provides additional phenotypic analysis of this mutant, the data do not yet support the author’s main conclusion that all of the changes are due to defects in GPI biosynthesis and GPI-protein assembly/trafficking. While the authors provide evidence that loss of GlcNAc6P-AT leads to a global defect in GPI, the study does not address the potential role of free versus protein-linked GPIs, or whether the concurrent loss of dolichol-oligosaccharides and/or protein N-glycosylation could also contribute to the phenotypes and parasite death. Further studies would therefore be needed to support the authors key conclusions and to progress the findings beyond what has been previously reported.

We thank the reviewer for their insightful comments. First, we would like to clarify that our study uses a rapamycin-inducible knockout system. While the relatively slow dynamics of rapamycin-mediated gene ablation may have caused some confusion, we emphasize that this system effectively and completely disrupts the *gna1* gene in the parasite genome.

In our previous works (Ref 5,6; [9,12]), we identified and characterized GNA1 in apicomplexan parasites, where it had not been previously uncovered due to its unique sequence features in this phylum, and we proved its essentiality for *P. falciparum* growth. However, these studies did not explore the specific downstream effects of enzyme disruption on parasite development, which is the primary focus of the current manuscript.

While GPI anchors are generally considered essential for *P. falciparum* growth, our study, along with the recent work by Bulloch et al. [1], is among the only evidence directly demonstrating how GPI depletion affects parasite development. Bulloch's study suggests that the absence of dolichol leads to GPI depletion, but does not fully dissect whether related alterations in lipid metabolism might also contribute to the observed phenotype. Our findings support the phenotype described by Bulloch *et al.*, further demonstrating that the observed defects are largely driven by alterations in GPI synthesis.

Finally, regarding the potential contribution of dolichol-oligosaccharides and/or *N*-glycosylation to the observed phenotypes, we hope the responses provided below to the Major Issues raised will help clarify that the alteration of GPI biosynthesis pathway is the primary cause of the observed phenotype, with minimal contribution from other UDP-GlcNAc-dependent pathways.

**Major Issues: Key Experiments Required for Acceptance**

Fig 1F. The signal for the HA-tagged protein in the immunoblot is very low/indistinct and it is hard to assess whether HA-protein is still expressed in the rapamycin-treated parasites (in which there is no background signal). Importantly, the Commassie Blue stained gels - which are used as a loading control but appear to be from different blots - suggest that less protein was loaded of the rapamycin-treated parasites. Overall, these analyses leave open the possibility that residual GlcNAc6P-AT activity may persist over more than one cycle. This is supported by the LC-MS data shown in Fig 2B-C which showed significant levels of UDP-GlcNAc and GlcNAc1/6P in the rapamycin-treated parasites. Further evidence demonstrating loss of GlcNAc6P-AT activity following knock-down and the kinetics of enzyme loss would assist with the interpretation of the other phenotypic analyses.

As the reviewer notes, the signal for the *Pf*GNA1-HA protein in the immunoblot is weak despite extensive optimization attempts (see our response to Reviewer 3 above), including testing various lysis and membrane transfer buffers, immunoprecipitation methods, and complementary approaches such as immunofluorescence. However, despite these efforts, we were unable to achieve a stronger signal. We also clarify that the Coomassie Blue-stained lanes were run on the same gel fragment as the blotted lanes to minimize variability. While it may appear that less protein was loaded for the rapamycin-treated parasites, we do not believe this accounts for the variations observed in the immunoblot. Notably, this rapamycin-inducible knockout system results in loss of *gna1* through genomic excision, and ImageJ-based auto-adjustment of brightness and contrast (now included in the revised Fig. 1F.) further highlights the complete absence of HA signal in the rapamycin-treated lane. Moreover, the marked phenotype (*i.e.*, complete parasite arrest at cycle 1 schizont stages) strongly suggests that any potential residual activity is negligible.

Regarding the LC-MS data in Fig. 2B-C, which shows significant levels of UDP-GlcNAc and GlcNAc1/6P in rapamycin-treated parasites and raises the possibility of residual GlcNAc6P-acetyltransferase activity persisting beyond cycle 0, we emphasize that metabolomic analysis was conducted on parasite-infected red blood cells enriched by magnetic cell separation (MACS). However, MACS retains a small fraction of uninfected RBCs, which naturally contain elevated pools of sugar nucleotides, markedly higher than those in parasites [13]. Additionally, residual metabolites in infected RBCs may also contribute to the detected baseline, further influencing the measured levels and explaining the residual UDP-GlcNAc and GlcNAc1/6P observed in rapamycin-treated samples. Therefore, rather than indicating sustained *Pf*GNA1 activity, these metabolites are likely the result of carry-over from red blood cells.

Finally, our analysis of parasite survival in cycles 0 and 1, together with the GlcNAc rescue experiments and the striking reduction of *Pf*GNA1 mRNA upon Rapamycin treatment introduced, strongly supports the conclusion that GlcNAc6P-acetyltransferase activity is virtually absent beyond cycle 0 (see below for further details).

The authors conclude that parasite death (and all other preceding phenotypic changes) primarily reflect loss of GPI-protein expression/trafficking, although direct evidence for this conclusion is not provided. In Fig 3A, the authors show that total GPI levels have decreased in rapamycin-treated parasites - however, it is not clear when parasites were harvested (cycle, time point) and to what extent loss of GPIs tracks loss of GlcNAc6P-AT activity. The GC-MS assay also does not distinguish between free GPIs and protein-linked GPIs. Further dissection of dynamics of GPI loss versus loss of UDP-GlcNAc synthesis and parasite death are needed to support the authors conclusions.

We thank the reviewer for their thoughtful comments and apologize for not clearly indicating the time point at which samples were collected for GPI quantification. Parasite samples used in the original Fig. 3A were harvested at the schizont stage (~40 hours post-invasion) at the end of cycle 1. At this stage, rapamycin-treated parasites retained only ~10% of total GPI levels relative to DMSO-treated controls.

To address the reviewer’s question regarding the relationship between GPI loss and GlcNAc6P-AT activity, we note that PCR analysis showed complete excision of the *gna1* gene by 40 hours post-treatment with rapamycin, corresponding to schizonts of cycle 0 (original Fig. 1C). In parallel, we have now performed RT-qPCR analysis of *gna1* mRNA using 25–30 hpi trophozoites from both cycles 0 and 1. This revealed a sharp decrease in transcript abundance in cycle 0 which is even more pronounced in cycle 1 (Supplementary Fig. S3; see page 5, line 138-144). These transcriptional changes correlate with a new GPI quantification performed on schizonts at 40 hpi in cycle 0 (Supplementary Fig. S10), where we observed a reduction in GPI levels in rapamycin-treated parasites, albeit less pronounced than in cycle 1 (Fig. 3A), when *gna1* is completely absent and the UDP-GlcNAc pool is likely depleted further. This new GPI quantification in cycle 0 has now been reported in the text (page 10, lines 233-236).

Additionally, HPLC-MS quantification of UDP-GlcNAc and GlcNAc1/6-P in 32–37 hpi trophozoites from cycle 1 showed significantly reduced levels compared to controls (original Fig. 2B and 2C). Finally, the marked ~90% reduction in total GPI levels in cycle 1 schizonts coincides with major defects in MSP1 processing and failed egress—hallmarks of impaired GPI-anchored protein function (original data).

While we acknowledge the challenge of directly linking parasite death and preceding phenotypes to GPI-protein expression and trafficking, the cumulative data strongly support that the severe GPI loss observed at 40 hpi in cycle 1 reflects the complete loss of GlcNAc6P-acetyltransferase activity and underlies the subsequent defects in parasite development and viability.

As noted by this and other reviewers, we cannot directly determine whether the observed phenotype arises from the loss of GPI-anchored proteins, free GPI, or both. However, the striking depletion of total GPI molecules in rapamycin-treated parasites strongly suggests that both are severely affected. Given the complexity of distinguishing these contributions, we consider this beyond the scope of the current study, but are designing future experiments to address this question in a follow up publication.

The authors suggest that the phenotypes observed following rapamycin-treatment are not due to loss of N-glycosylation, as these effects would only manifest in the second or later cycles. However, I am not aware that this has been demonstrated definitively in the literature and also that it is likely to be very dependent on the mutant studied (for example defects in isoprenoid synthesis have pleiotropic effects). It is also worth noting that disruption of the function of just one protein due to a partial loss of N-glycosylation may be sufficient to cause parasite death. As a result, the authors really need to confirm that N-glycosylation is not affected in the first cycle and that hypo-N-glycolsylation of one or more proteins does not underlie the observed phenotypes.

We acknowledge the ongoing debate regarding the role of *N*-glycosylation in *P. falciparum*. To address the reviewer’s insightful concern and to distinguish the effects of *N*-glycosylation inhibition from those of HBP disruption, we treated asexual blood-stage parasites with tunicamycin, a well-established and specific inhibitor of UDP-N-acetylglucosamine:dolichyl-phosphate N-acetylglucosaminephosphotransferase (PF3D7_0321200). By inhibiting this enzyme [14], tunicamycin effectively disrupts *N*-glycosylation in asexual blood-stage parasites [15]. Crucially, tunicamycin does not impair parasite growth during the cycle of treatment. Instead, it induces a delayed death phenotype, where parasites appear normal in the treated cycle but fail to survive in the next, dying at trophozoite stages. Notably, this delayed death phenotype is not related to the isoprenoid precursor synthesis [15].

Unlike tunicamycin treatment, disruption of *Pf*GNA1 and the HBP causes parasite death at the schizont stages, when GPI biosynthesis is at its height. The timing and dynamics of rapamycin-induced DiCre-mediated gene disruption adds complexity to its interpretation: while gene disruption is triggered at the beginning of cycle 0, complete gene excision is achieved at the end of this cycle and its full effect manifests later in cycle 1. We acknowledge that this may cause some confusion regarding the different modes of action and the specific pathways involved, and have endeavored to clarify this further in the text (page 5, lines 138-144).

Therefore, to rule out concerns about the impact of UDP-GlcNAc depletion during cycle 0, we supplemented the media with GlcNAc at this initial cycle. This ensures that both DMSO- and rapamycin-treated parasites experienced comparable conditions during cycle 0 (Supplementary Fig S8B and C), isolating the effects of gene disruption to cycle 1. When GlcNAc was removed just prior to the transition from cycle 0 to cycle 1, rapamycin-treated (*Pf*GNA1-disrupted) parasites failed to progress beyond the schizont stage in cycle 1, which aligns with the phenotype of schizont-stage death and failed egress due to GPI biosynthesis impairment (Supplementary Fig S8D and E). These results support our hypothesis that the HBP-GPI axis is a primary driver of the observed phenotype, even though it does not preclude minor effects from other, less well-characterized or potentially unknown pathways.

With this experimental setup established, we first rescued parasites with GlcNAc throughout cycle 0. After removing GlcNAc, we then added tunicamycin in cycle 1. In the controls (DMSO + tunicamycin), parasites exhibited the signature delayed death phenotype caused by tunicamycin, stalling at the trophozoite stage in cycle 2. In striking contrast, rapamycin-treated parasites (both, + and – tunicamycin) were unable to progress beyond cycle 1 and stalled as cycle 1 schizonts. This confirms that both HBP disruption and UDP-GlcNAc depletion induce parasite death within the same cycle as detailed in Supplementary Fig S9. To clarify these aspects, the experimental evidence has been reported in the manuscript (page 10, line 230-233) and included as Supplementary Fig. S8 and S9.

These findings indicate that parasite death in cycle 1 results from HBP disruption, leading to UDP-GlcNAc depletion and GPI reduction rather than any *N*-glycosylation defects. Our data strongly support that HBP disruption and UDP-GlcNAc depletion are lethal before any delayed death effects linked to *N*-glycosylation inhibition. While UDP-GlcNAc depletion may affect *N*-glycosylation, if this process contributed to the observed phenotype, its effects would emerge in the next cycle, with parasite death occurring at the trophozoite stage, which is consistent with the delayed death hallmark of *N*-glycosylation inhibition. In contrast, we observe parasite death in schizonts within the same cycle, reinforcing that HBP disruption and UDP-GlcNAc depletion drive GPI decline as the primary lethal event. These experiments carried out strengthen the study conclusions by clearly dissecting the dominant role of GPI biosynthesis defects over *N*-glycosylation impairment in driving parasite death. Furthermore, the strong phenotype observed, where no parasites survive, highlight the importance of UDP-GlcNAc as a major bottleneck metabolite for parasite survival.

Previous studies have shown that knock-down of GlcNAc6P-AT can be biochemically by-passed by supplementing cultures with GlcNAc. Inclusion of this control would provide strong support for the function of GlcNAc6PAT being primarily/exclusively enzymatic, rather than non-enzymatic, and should be included.

We appreciate this comment and have now included the suggested control. To assess whether the function of *Pf*GNA1 is primarily enzymatic, we supplemented the culture with GlcNAc at the beginning of cycle 1, 48 hours after rapamycin treatment. This timing was chosen to allow for maximal protein degradation and depletion of the UDP-GlcNAc pools, given the dynamics of DiCre-mediated gene disruption. The new data specifically demonstrate that GlcNAc supplementation rescues the phenotype observed in *Pf*GNA1-disrupted parasites after complete gene knockout. Whereas rapamycin-treated parasites (*Pf*GNA1-disrupted) fail to progress beyond the schizont stage and die in cycle 1, GlcNAc-supplemented parasites grow at the same rate as DMSO-treated (non-disrupted) controls. These findings strongly support that *Pf*GNA1’s essential function in asexual blood-stage parasites is enzymatic, rather than non-enzymatic. These data have now been included as Supplementary Fig. S4, and the manuscript modified accordingly (page 6, lines 152-155).

**Minor Issues: Editorial and Data Presentation Modifications**

Do the authors have any thoughts on why other metabolites unrelated to hexosamine synthesis (e.g N-carbamoyl-Asp) are elevated in the knock-out?

The increase of N-carbamoyl-L-aspartate, a metabolite associated with pyrimidine metabolism, in rapamycin-treated parasites remains unexplained. Similarly, we observed increased levels of dUMP, a pyrimidine biosynthesis intermediate, and 5-phosphoribosyl-1-pyrophosphate (PRPP), a central metabolite in purine and pyrimidine nucleotide synthesis. One hypothesis could be that HBP disruption could drive UTP accumulation. UTP, a pyrimidine metabolism intermediate, is required for converting GlcNAc-1P to UDP-GlcNAc, a process hindered by HBP inactivation. This disruption might subsequently perturb other pyrimidine metabolism intermediates, such as those observed here. However, these changes could also reflect broader metabolic disturbances linked to rapamycin-induced growth impairment and parasite death. We (Llinás lab) have previously seen significant perturbation (likely non-specific) of pyrimidine biosynthesis by a large array of compounds which are effective at killing malaria parasites [16,17]. Therefore, it appears that significant modulation of pyrimidine metabolism is strongly associated with parasite death. Given the speculative nature of these connections, we avoid overinterpreting the metabolomic data without further mechanistic validation.

**References**

1.	Bulloch MS, Huynh LK, Kennedy K, Ralton JE, McConville MJ, Ralph SA. Apicoplast-derived isoprenoids are essential for biosynthesis of GPI protein anchors, and consequently for egress and invasion in Plasmodium falciparum. PLoS Pathog. 2024;20: e1012484. doi:10.1371/JOURNAL.PPAT.1012484

2.	Ramaprasad A, Burda PC, Calvani E, Sait A, Palma-Duran SA, Withers-Martinez C, et al. A choline-releasing glycerophosphodiesterase essential for phosphatidylcholine biosynthesis and blood stage development in the malaria parasite. Elife. 2022;11. doi:10.7554/ELIFE.82207

3.	Koussis K, Withers-Martinez C, Baker DA, Blackman MJ. Simultaneous multiple allelic replacement in the malaria parasite enables dissection of PKG function. Life Sci Alliance. 2020;3. doi:10.26508/LSA.201900626

4.	Patel A, Fréville A, Rey JA, Flynn HR, Koussis K, Skehel MJ, et al. Plasmodium falciparum protein phosphatase PP7 is required for early ring-stage development. mBio. 2024;15: e0253924. doi:10.1128/MBIO.02539-24

5.	Boonyalai N, Collins CR, Hackett F, Withers-Martinez C, Blackman MJ. Essentiality of Plasmodium falciparum plasmepsin V. PLoS One. 2018;13. doi:10.1371/JOURNAL.PONE.0207621

6.	Thomas JA, Tan MSY, Bisson C, Borg A, Umrekar TR, Hackett F, et al. A protease cascade regulates release of the human malaria parasite Plasmodium falciparum from host red blood cells. Nature Microbiology 2018 3:4. 2018;3: 447–455. doi:10.1038/S41564-018-0111-0

7.	Farrell B, Alam N, Hart MN, Jamwal A, Ragotte RJ, Walters-Morgan H, et al. The PfRCR complex bridges malaria parasite and erythrocyte during invasion. Nature. 2024;625: 578–584. doi:10.1038/S41586-023-06856-1

8.	Robert-Paganin J, Robblee JP, Auguin D, Blake TCA, Bookwalter CS, Krementsova EB, et al. Plasmodium myosin A drives parasite invasion by an atypical force generating mechanism. Nat Commun. 2019;10. doi:10.1038/S41467-019-11120-0

9.	Chi J, Cova M, De Las Rivas M, Medina A, Junqueira Borges R, Leivar P, et al. Plasmodium falciparum apicomplexan-specific glucosamine-6-phosphate n-acetyltransferase is key for amino sugar metabolism and asexual blood stage development. mBio. 2020;11: 1–15. doi:10.1128/MBIO.02045-20

10.	Paneque A, Fortus H, Zheng J, Werlen G, Jacinto E. The Hexosamine Biosynthesis Pathway: Regulation and Function. Genes (Basel). 2023;14. doi:10.3390/GENES14040933

11.	Wellen KE, Lu C, Mancuso A, Lemons JMS, Ryczko M, Dennis JW, et al. The hexosamine biosynthetic pathway couples growth factor-induced glutamine uptake to glucose metabolism. Genes Dev. 2010;24: 2784–2799. doi:10.1101/GAD.1985910

12.	Cova M, López-Gutiérrez B, Artigas-Jerónimo S, González-Díaz A, Bandini G, Maere S, et al. The Apicomplexa-specific glucosamine-6-phosphate N-acetyltransferase gene family encodes a key enzyme for glycoconjugate synthesis with potential as therapeutic target. Sci Rep. 2018;8: 4005. doi:10.1038/s41598-018-22441-3

13.	López-Gutiérrez B, Dinglasan RR, Izquierdo L. Sugar nucleotide quantification by liquid chromatography tandem mass spectrometry reveals a distinct profile in Plasmodium falciparum sexual stage parasites. Biochemical Journal. 2017;474: 897–905. doi:10.1042/BCJ20161030

14.	Yoo J, Mashalidis EH, Kuk ACY, Yamamoto K, Kaeser B, Ichikawa S, et al. GlcNAc-1-P-transferase–tunicamycin complex structure reveals basis for inhibition of N-glycosylation. Nature Structural & Molecular Biology 2018 25:3. 2018;25: 217–224. doi:10.1038/s41594-018-0031-y

15.	Fenollar À, Ros-Lucas A, Pía Alberione M, Martínez-Peinado N, Ramírez M, Ángel Rosales-Motos M, et al. Compounds targeting GPI biosynthesis or N-glycosylation are active against Plasmodium falciparum. Comput Struct Biotechnol J. 2022;20: 850–863. doi:10.1016/J.CSBJ.2022.01.029

16.	Antonova-Koch Y, Meister S, Abraham M, Luth MR, Ottilie S, Lukens AK, et al. Open-source discovery of chemical leads for next-generation chemoprotective antimalarials. Science (1979). 2018;362. doi:10.1126/SCIENCE.AAT9446,

17.	Allman EL, Painter HJ, Samra J, Carrasquilla M, Llinás M. Metabolomic profiling of the Malaria Box reveals antimalarial target pathways. Antimicrob Agents Chemother. 2016;60: 6635–6643. doi:10.1128/AAC.01224-16,
